# Relevance of Milk Composition to Human Longitudinal Growth from Infancy Through Puberty: Facts and Controversies

**DOI:** 10.3390/nu17050827

**Published:** 2025-02-27

**Authors:** Katarina T. Borer

**Affiliations:** School of Kinesiology, The University of Michigan, Ann Arbor, MI 48104, USA; katarina@umich.edu

**Keywords:** breast milk, dairy animal milk, formula milk, milk composition, milk bioactivities, longitudinal growth, infant gut metabolome, obesity, metabolic dysfunction, critical nutritional periods

## Abstract

Milk is the principal nutrient of newborn humans and a diagnostic feature of the order Mammalia. Its release is elicited as a reflex by infant sucking under the control of the hormone oxytocin. While it is recognized that breast milk optimally promotes infant longitudinal growth and development, this review explores facts and controversies regarding the extent to which the milks of several dairy animals and infant formula milk (IF) approximate special properties and bioactivities of breast milk. It also provides evidence that early exposure to undernutrition during the very rapid fetal and early infancy growth predominantly and permanently stunts longitudinal growth trajectory in both animals and humans and is often followed in later life by obesity and metabolic dysfunction, and sometimes also by precocious timing of sexual maturation. There is a knowledge gap as to whether there may be additional critical periods of nutritional vulnerability in human development, which is characterized by a relatively prolonged period of slow childhood growth bracketed by the rapid fetal–neonatal and pubertal growth spurts. It is also unclear whether any quantitative differences in caloric intake and supply during neonatal period may influence developmental fatness programming. A further knowledge gap exists regarding the role of infant microbiome composition and development in the possible epigenetic programming of longitudinal growth or fatness in later life. Extending the research of early developmental programming to the entire period of human growth from conception to the end of puberty, examining infant caloric intake and supply as possible factors modulating the epigenetic programming in favor of obesity, and examining the role of infant gut microbiome in developing infant’s capacity to process nutrients may provide a better understanding of the interaction between critical nutritional influences in the control of human longitudinal growth and later-life obesity.

## 1. Introduction

Milk is an evolutionary innovation refined by natural selection for optimal nutrition of newborn mammals, including human infants. The order Mammalia, to which humans and other mammals belong, developed during the Jurassic Mesozoic period, about 160 million years ago. The evolution of milk provided a more effective nutrient mix and mode of delivery for newborns than the older system of providing the nutrition within extruded shelled eggs, estimated to have begun 320 million years ago, which was used by dinosaurs and persists to the present day in birds, and crocodilians [1]. Two genetically programmed reflexes facilitate non-competitive availability of milk for infants for several months after birth. One, a milk ejection reflex, is mediated by the posterior pituitary hormone oxytocin (OXY) triggered from one group of hypothalamic neurons in response to infant sucking on the breast nipple [2]. Milk letdown can be conditioned by mother hearing infant crying or even thinking about nursing [3]. Interestingly, the same hormone is responsive to other kinds of skin contact, promoting social bonding between mother and infant or between humans generally [4]. The second reflex involves interference by breast feeding with pregnancy for up to 6 months following parturition provided the infant is nursing intensively [5]. This reserves access to ample milk supply to the newborn infant for the first 6 months of its life. Several health organizations acknowledge the presence of these reflexes and also advocate breast-feeding’s benefit to the infant during the 6 to 12 months after birth [6,7,8].

The next development regarding human milk consumption took place about 10,500 years ago, in Anatolia (the Asian portion of present-day Turkey), and possibly simultaneously in other parts of the world, when humans domesticated cattle, goats, sheep, and camelids [9,10], started fermenting kefir and yogurt, and developed the ability to drink raw milk. 

There is a wealth of evidence-based facts about the benefits of breast milk in promoting the growth and development of newborn infants [11], and their health in adulthood [12]. Despite the abundance of research on the capacity of this milk to stimulate growth and healthy development in breast-fed infants, and of milk and its products on health in children and adult humans, some unresolved questions remain. Several prominent ones are as follows: How does milk produced by cows, sheep, goats, and camels compare to human milk and synthetic infant formula (IF) in stimulating growth, body composition, and other aspects of health in infants, children, and adolescents? Are there critical periods of nutritional vulnerability that impair longitudinal growth and produce metabolic dysfunctions? Can a quantitative difference in caloric intake of breast-fed and IF-fed infants, exposure to hyperinsulinemia through breast milk, and post-deprivation catch-up growth, lead to programming of obesity in later life? Does the developing gut microbiome control the infant’s capacity to absorb nutrient energy toward longitudinal and ponderal growth? The purpose of this literature review is to address the facts and controversies regarding these questions.

## 2. Method of Search and Inclusion of Articles

Articles were searched in PubMed and Google Scholar using key words featured in the subsections of this review. The search emphasized recent reviews and meta-analyses, against which the individual studies were analyzed and evaluated. The focus was the effects of milk composition and bioactivity on human infant longitudinal growth, body composition, and defenses against infectious disease. Data from animal studies were not included. Accurate measurements of milk qualities are challenging because the composition of milk appears to change in response to the species of source animal, timing of milk analysis relative to the stage of lactation, and measurement methods. Because of measurement challenges and ethical issues, most studies assessing the role of milk composition on infant growth and development are observational, and their results represent correlations between associated variables rather than cause-and-effect evidence.

## 3. How Does Milk Produced by Cows, Sheep, Goats, and Camels Compare to Human Milk and Infant Formula in Stimulating Infant Longitudinal Growth and Affecting Body Composition?

Properties of the milk of different species of lactating mammals have evolved to support their specific growth rates and other biological functions.

A comparison of the chemical composition of human, cow, camel, goat, and sheep milk and infant IF milk can reveal the degree of their equivalence and value in serving as potential substitutes when human mothers cannot, or prefer not to, breast-feed their infants. While human milk contains about 87% water, 4% fat, 1% protein, and 7% lactose, it is a complex medium that also contains additional constituents, many of which interact with each other and confer to breast milk special bioactivities [13]. Major components of breast milk are broadly comparable in the five species of dairy animals (Table 1). Species differences are evident mostly in the concentrations of protein and fat. Human milk contains the least protein, sheep contains the most, and cow, camel, and goat have intermediate amounts. Sheep milk is richest in fat and in the ratio of whey protein to casein. Concentrations of lactose and most other components are less variable (Table 1).

Among dairy breeds of domesticated animals, goat milk is the second most widely produced and consumed milk after cow milk (Wikipedia). In addition to the Mediterranean countries (France, Spain, Turkey), including the blue longevity zone of Italian Sardinia, Netherlands and Eastern Europe are also producers and consumers of goat milk. Goat milk is also consumed in the Middle East, and Asia (India, Pakistan, Bangladesh, and China). India accounts for 25% of goat milk production and consumption. Feta is best known among the 30-plus varieties of goat cheese. Sheep milk is also produced and consumed in the Mediterranean, Europe (particularly France), and Pacific Asia. Among the great variety of cheeses made from sheep milk are Italian ricotta, French Roquefort, and Croatian pag cheese. Camel milk is consumed in Africa, India, Pakistan, Mongolia, and Middle Eastern countries. It has for millennia provided important nutrition to Middle Eastern nomad populations in desert climates. Out of 2.8 million tons of camel milk produced and consumed in 2017, Somalia and Kenya contribute 30% to 33% each. Between 2014 and 2017, Australia and US had 600,000 and 5000 camels, respectively, providing locally consumed milk and milk products. Camel’s milk has a similar taste to cow’s milk, but its properties make it difficult to make cheese with. Though all reviewed papers regarding non-human dairy animal milks [14,15,16,17,18,19,20,21,22] provided compositional information, none documented the effects on infant growth and development, and only one focused on camel’s milk digestibility for infants and on its benefits in counteracting the adult risk of type 2 diabetes and coronary heart disease [14].

**Table 1:** Composition of human milk [13] relative to animal milk substitutes: cow [13], camel [19,20], goat [21,22], and sheep milk [22].

Milk Components	Human	Cow	Camel	Goat	Sheep
Protein (g/100 mL)	1.0	3.4	3.4	3.6	5.6
Casein (g/100 mL)	0.4	2.7	2.4	2.1	4.2
α_s1_-casein (*w*/*w*)	0	1.0	0.5	0.1	0.5
α_s2_-casein (*w*/*w*)	0	0.3	0.2	0.4	0.4
β-casein (*w*/*w*)	0.3	1.0	1.6	1.2	3.2
κ-casein (*w*/*w*)	0.1	0.4	0.1	0.4	0.1
Whey proteins (g/100 mL)	0.7	0.6	0.7	0.6	1.1
Fat (g/100 mL)	4	4.4	3.3	4.5	7.5
Saturated fatty acids (SFA) (*w*/*w*)	2	3.2	2.4	2.7	4.6
Monosaturated (MUFA) (*w*/*w*)	1.7	1.1	0.8	1.1	1.7
Polyunsaturated (PUFA) (*w*/*w*)	0.5	0.2	0.1	0.2	0.3
Cholesterol (mg/100 mL)	13	13	6	11	22
Emulsified fat globules (μm) (*w*/*w*)	2.5	4	3	3	4
Carbohydrates: lactose (g/100 mL)	6.7	5.5	4.3	4.3	4.6
Kilocalories/100 mL	71	61	78	69	108

*w*/*w* = grams of specific fat or protein.

### 3.1. Properties of Human Milk

The composition of human milk appears to change in response to many variables, such as the time relative to onset of lactation, the course of individual feeding, and the interval since last fed [23]. Characteristics of the mother, such as her age, diet, and BMI, also play a role [24], as well as infant’s gender [25] and infant’s requirements as they change with age. With respect to stage of lactation, breast milk composition changes from colostrum during the first seven days after birth to transitional milk (eighth to 15th day after birth), and then to mature milk subsequent to the 16th day after birth [26,27]. During the transition to mature milk, lactose peaks by the seventh month, and the concentration of lipids progressively increases, while high concentrations of growth factors found in the colostrum decline [24]. In the course of a single nursing bout, fat content increases, and lactose decreases between foremilk and hindmilk [28]. In young mothers between 20 to 30 years, breast milk contains the highest concentration of protein but does not affect lipids and lactose. Maternal dietary fatty acids (6-omega vs. 3-omega) appear in breast milk within 2 to 3 days after birth [29], while breast tissue can synthesize medium-chain fatty acids (MCFAs) of between 10 and 14 carbons.

#### 3.1.1. Colostrum

Colostrum is the first milk delivered to the sucking newborn during the first week after birth [26]. It is different from mature milk in that it contains very high concentrations of whey protein rich in immunoglobulin IgA, has little fat and lactose, and an almost undetectable concentration of casein. The absence of casein, but high concentrations of immunoglobulins, and double the concentration of oligosaccharides (HMOs) compared to mature milk, suggest that the principal function of colostrum is for immunologic protection of the baby outside the mother’s sterile uterine environment. [24,30]. In addition to its nutritional and immunological role, colostrum strongly promotes growth, as it is richer in growth factors, such as epidermal growth factor, TGF-β, and colony stimulating factor, than mature milk [24].

#### 3.1.2. Milk Protein

Human breast milk contains approximately one third of the amount of protein found in cow, camel and goat milk, and even less compared to sheep milk. Milk proteins include caseins, whey, and mucins [13]. Whey proteins occur in the soluble phase of milk. Their concentration in human milk is 30% higher than that of casein while, in cow, camel, and goat milk, casein concentration is between 3.5 and 4.5 times higher than whey protein [10]. The whey/casein ratio in human milk fluctuates between 80:20 and 70:30 in early lactation and decreases to 50:50 in late lactation [31]. Caseins form suspended micelles. Three types of casein are present in human milk: α-, β-, and k-casein. K-casein forms a colloidal micelle, a suspended protein clump containing α-, and β-caseins. Mucins occur in the membrane of milk-fat globules. Whey proteins include α-lactalbumin, lactoglobulin, lactoferrin, serum albumin, lysozyme enzymes, and immunoglobulins. like secretory IgA. Other whey proteins are folate-binding protein, bifidus factor, micronutrient digesting enzymes amylase, lipase, α1-antitrypsin and anti-chymotrypsin, and protein B12-protecting haptocorin [13,24,31]. A number of these proteins have special bioactive functions [31]. Alpha-lactalbumin controls lactose synthesis and binds Ca and Zn ions. Casein micelles bind calcium and phosphorus. Lactoferrin and lysozyme have antibacterial functions, while the immunoglobulin IgA destroys bacteria and protects infant’s intestinal mucosa. The breakdown products of milk proteins, peptides and amino acids, provide the building blocks for infant growth. Breast milk also contains growth factors and hormones imported from maternal blood, molecules derived from maternal and infant gastrointestinal microbiome, vitamins, minerals, and immune and stem cells [31].

#### 3.1.3. Milk Fat

Lipids contribute between 40% and 55% of total breast milk energy [14]. Milk fat exists in the form of an emulsion. Over 95% of lipids exist as triglycerides which, along with diglycerides, monoglycerides, free fatty acids (FAAs), phospholipids, and cholesterol, are packaged into milk fat globules. The sphingomyelins in the coating of fat globules support the development of infant lungs and brain myelinization. Some FAs (with 8 to 12 carbon chains) are synthesized de novo in the mammary cells. Other, both short chain (up to 6 carbons), and longer chain FAs, are formed from circulating metabolites, including those produced by fermentation of carbohydrate by rumen bacteria. Others are produced in some organs, and all are carried to the mammary gland via lipoproteins. The most plentiful FAs in breast milk are monounsaturated oleic (18 carbons, 36% of FAs), saturated 16-carbon palmitic (27%), and 18-carbon polyunsaturated linoleic (15%) acids. Two 18-carbon FAs, alpha linolenic (omega 3) and linoleic (omega 6), are essential and must be obtained from the maternal diet. They are converted to a 20-carbon arachidonic acid (AA, omega 6) and eicosapentaenoic acid (EPA, omega 3), while EPA is converted to a 22-carbon docosahexaenoic acid (DHA, omega 3). AA, EPA, and DHA are important for supporting infant growth and immune system. They mediate inflammatory responses and support infant neuromotor and sensory development [31].

#### 3.1.4. Milk Carbohydrates

Lactose is the main carbohydrate in the milk of all examined mammals. It is a disaccharide composed of glucose and galactose (galactose-β1,4-glucose) produced by lactose synthetase in the mammary gland. Breakdown of the unusual β1,4 disaccharide bond is performed by the lactase enzyme (also known as β-galactosidase or lactase phlorizin hydrolase) produced by the infant small intestine, which frees up contained monosaccharides. The lactose disaccharide bond is, in part, responsible for the stability of milk osmotic pressure in view of changes in concentrations of fat and protein in the course of a single nursing episode, or in the stages of infant development [31]. Galactose derived from lactose supports infant liver glycogen development. In addition to providing 40% of nutrient value for the infant per deciliter of milk, in the form of glucose and galactose, additional lactose benefits are its low glycemic index (GI = 46) due to a lack of insulin response to galactose, and an absence of stimulation of the brain reward centers, due to its low sweetness [32]. Glucose polymers, like digestible maltodextrins, formed by 1–4- and 1–6-linked glucose molecules, often serve as lactose substitutes in IFs. Maltodextrins promote rapid elevation of blood glucose, have a glycemic index of 110 [33], and are significantly sweeter than lactose [32]. This is relevant to the development of the taste buds and olfactory receptors during third trimester of pregnancy and a strong preference of newborns for a sweet taste [34]. Early infant exposure to the rewarding sweet taste in lactose-free infant formulas may predispose children and adolescents to overconsumption of brain-rewarding sweet food [35] and to obesity [36]. Maltodextrins may also facilitate protein glycation, a bond between sugar and protein amino groups. Protein glycation, in turn and over time, generates advanced glycation end products (AGEs) that contribute to diabetes and metabolic disorders. Any undigested lactose that reaches the terminal ileum and colon stimulates fermentation by microbiota and colonization by *Bifidobacteria* and other beneficial lactic acid bacteria. Lactose also induces colonization of the infant GI tract by anti-microbial bacteria.

In populations where the expression of lactase enzyme gene LAC is deficient, lactose malabsorption and intolerance syndromes are observed. Globally, between 65% and 70% of the population are lactose intolerant or lactase-nonpersistent. It is estimated that the evolution of the constitutive lactase gene LAC in approximately 30% to 35% of Europeans and Africans occurred convergently between 7000 and 5000 years ago, shortly after domestication of dairy animals, because of the selective nutrient advantage of milk and its products [37]. Before the lactose tolerance evolved, fermented milk products were probably initially consumed because of their lower concentration of lactose.

Besides lactose, milk carbohydrates include several hundred different oligosaccharides or HMOs comprising about 1.3% of mature milk and about 2% at day 4 after birth [24]. Over 200 characterized HMOs contain different combinations of five different sugars (D-glucose, D-galactose, L-fucose, sialic acid, and N-acetylglucosamine), in addition to lactose. HMOs act as probiotics facilitating the growth of beneficial bacteria (bifidobacteria and lactobacilli) and help establish infant intestinal microbiota. HMOs defend infants against pathogens, like *Campilobacter diarrheae*, *Streptococcus pneumoniae* and *Escherichia coli*, by acting as decoys, preventing binding of pathogenic bacteria to the infant’s intestinal wall.

#### 3.1.5. Milk Vitamins and Minerals

Human milk contains all the metabolically important water-soluble vitamins, including C and nine B vitamins. It also contains fat-soluble A and E vitamins but is relatively low-to-deficient in K and D vitamins [29]. At concentrations of 0.15, 0.37, 2.1, and 0.1 mg/L, vitamins B1 (thiamin), B2 (riboflavin), B5 (pantothenic acid), and B6 (pyridoxine) are 35%, 22%, 58%, and 21% lower, respectively, in human milk than in cow’s milk. However, human milk has 85% more of B3 (niacin, 1.7 mg/L), and twice as much B8 (inositol, 300 mg/L) as cow’s milk, while concentrations of B12 (cobalamin) and biotin are very low in both (<0.005 mg/L). Of the fat-soluble vitamins, human milk has 43% more A (0.53 mg/L), about the same amount of its precursor carotene (0.24 mg/L), and about 5 times more E (tocopherol, 5.4 mg/L) than cow’s milk, while the concentrations of vitamin K (phylloquinone) and of vitamin D (cholecalciferol) are low in both (0.02 and <0.001 mg/DL). Near absence of vitamin D in human and cow’s milk is of concern because of its importance in calcium absorption, bone mineralization, and neuromuscular and immune defense functions. Its deficiency can cause rickets in infants. During pregnancy, vitamin D appears to perform primarily an immune function rather than regulating skeletal homeostasis. It is speculated that the human behavioral change regarding wearing clothing and using sunscreens has reduced ancestral exposure to solar energy, which normally stimulates vitamin D formation. This change has occurred too recently to trigger an evolutionary compensatory adaptation. To prevent rickets, supplementation with 400 IU daily for the first year of an infant’s life is recommended [38].

Minerals in human milk are an essential part of many important enzymes and contribute to structural components of the infant body [13]. Five of the macro-minerals are present at lower concentrations in human milk than in cow’s milk. Phosphorus (130 mg/L) makes up 13% of the concentration of cow’s milk. Calcium and magnesium at 300 and 30 mg/L, respectively are 25% of human relative to cow’s milk, while potassium and chloride at 600 and 430 mg/L are, respectively, 40% and 45% of human compared to cow’s milk. The trace minerals iron, zinc, copper, manganese, fluoride, selenium, cobalt, chromium, and molybdenum are represented in similar concentrations of between 1 μg to 1.6 mg in human and cow’s milk, with the exception of iodine, which is present at 27% (70 μg) in human relative to cow’s milk.

#### 3.1.6. Milk Hormones

The foremost involvement of hormones in breast feeding is that milk secretion is initiated by the exclusively mammalian OXY. Milk let-down reflex is mediated by supraoptic and paraventricular hypothalamic neurons, which release OXY from the posterior pituitary into systemic circulation in response to infant sucking stimulation [2,39]. This triggers rhythmic contractions of mammary ducts in both breasts as long as the infant sucks [40]. Besides its control of milk ejection, OXY also assists in parturition through elicitation of uterine contractions during childbirth [2]. A second set of OXY projections terminates in the bed nucleus of stria terminalis in the medial basal forebrain and in the ventromedial hypothalamus, brain regions responsive to sex steroids [41]. When activated by OXY, these neurons facilitate social and prosocial behaviors, such as attachment and bonding of infants with mother, between animals [41] and between humans [42]. The role of OXY in bond formation and pro-social behaviors appears to be, like sucking behavior in milk ejection, mediated by body touch [4].

Sucking behavior after the infant’s birth, rather than OXY, suppresses pregnancy for 6 months, corresponding to the lactation period recommended by health agencies [6,7,8]. The effect appears to be mediated by the duration and frequency of sucking episodes, which need to last greater than 60 min and take place more than 5 times per day [5]. Such nursing blocks menstruation by interfering with follicle development and ovulation, as it alters the pattern of gonadotropin, and increases prolactin, secretion [5].

There is also intense interest in the hormonal composition of breast milk obtained from maternal circulation and its possible influence on infant growth and development.

The list of hormones and cytokines described in human milk includes apelin, beta-endorphin, cholecystokinin, cortisol, estrogen, ghrelin, glucagon-like peptide 1 (GLP-1), insulin, insulin-like growth factor 1 (IGF-1), irisin, leptin, melatonin, motilin, neuropeptide Y, obestatin, peptide YY, progesterone, resistin, thyroid hormones, etc. [24,43,44,45,46,47,48,49,50,51]. The significance of most of these hormones in human milk for infant growth and body composition, if any, is not yet fully understood. Beta endorphin was associated with infant crying, colic, and maternal disturbed sleep [44]. Progesterone, but not estrogen, concentration was negatively related to maternal protein intake [48]. Melatonin rhythm in maternal circulation and in breast milk were synchronized, suggesting that circadian timing of breast feeding may be important [45]. Triiodothyronine (T3), but not thyroxin (T4), was detected in breast milk [49]. Myokine irisin concentration did not vary between different stages of milk secretion [50], while early morning circadian elevations and midnight nadir for cortisol had no apparent effect on infant behavior [48].

Adipokines leptin, adiponectin, obestatin and resistin in human breast milk have attracted research because these cytokines are released from the adipose tissue and are therefore assumed to potentially influence body composition and development of fat tissue in later life [43,47]. Research on adipokine leptin and hormone insulin is justified as they are implicated in the control of appetite and body composition in adulthood. The working hypothesis is that these messengers may cause developmental programing of infant’s appetite and weight gain toward, or away from, obesity in later life. Justification for interest in leptin was a demonstration that humans who are genetically unable to produce leptin experience intense hunger and display a high level of obesity, manifestations that can be abolished with leptin administration [52,53]. Leptin inhibits orexigenic neurons in the hypothalamus [54]. It also increases lipolysis and fat utilization and facilitates hunger when its concentrations decline during energy restriction [54,55]. Insulin is the key hormone controlling nutrient intake and storage in the form of inert molecules of glycogen, fat, and structural proteins [56]. The two messengers actually form a counterregulatory team in adulthood [57], in that meal-induced insulin secretion facilitates leptin secretion from the subcutaneous adipose tissue, and leptin, in turn, suppresses insulin secretion and actions, while promoting lipolysis.

There is a good deal of controversy regarding the hypothesis for leptin-insulin developmental programming during infancy. Two reviews present contrasting evidence, with one mostly listing the inconsistencies and contradictions [58], and the other presenting supportive evidence [59] for the hypothesis. In a recent systematic review [60], concentrations of adipokines were compared to several parameters of infant growth. The results, usually measured over less than a year of infant life, were mostly inconsistent, and at times contradictory. Adiponectin was inversely proportional to infant fat in three studies, positively related to fat in one, inversely related to longitudinal growth in one, and not associated with any change in three studies. Leptin in human milk did not affect body composition in four studies, of which one had a 3- and 5-year follow-up. It was also associated with four studies reporting increases in fat mass or body weight and in three that reported the reverse outcome. Ghrelin and insulin were both associated with infant weight and weight-to height gain in five studies and not associated with any body composition variable in two. Despite the inconsistent associations in the above studies between insulin and leptin concentrations in infant milk and anthropometric outcomes, there is a significant correlation between high insulin and leptin concentrations and the obesity of lactating mothers [61]. Thus, hyperinsulinemia and hyperleptinemia in the milk of obese mothers, which reflects their insulin and leptin resistance, may possibly expose their infants to developmental programming of obesity in later life.

The final issue is a relative silence in the literature on human milk regarding the potential role of growth hormones on perinatal infant growth, as growth hormone (GH) regulates longitudinal growth in childhood and adolescence through the mediation of the hormone IGF-1 and its binding proteins [62]. Absence of GH measurements in milk-hormone studies may partially reflect the inconvenience of the nocturnal timing of most GH secretion, and the difficulty of frequent blood or breast milk sampling to capture the defining mechanism of stimulation of growth through the changes in GH pulsatility [63]. IGF-1 has been detected in breast milk [46], where its concentration, and that of leptin, was correlated with rapid infant growth at 3 months. IGF-1 concentrations and its binding proteins were higher in preterm than in term human milk [64] and at higher concentrations during the first week of life than in later weeks, when they are postulated to stimulate the maturation of the infant gut [65]. None of these studies evaluated the relevance of the measurements to infant linear growth or body composition.

#### 3.1.7. Milk Enzymes

The properties and composition of human milk have been presented to this point as being stable and predictable milk constituents throughout the period of lactation. This turns out to be incorrect, as recent advances in mass spectrometry and in peptidomic and bioinformatic methods reveal that enzymatic breakdown of nutrients creates a complex dynamic collaborative system. The enzyme components in mother’s milk and infant organs are integrated into a specific, appropriately timed, program that includes pre-digestion of milk proteins in mother’s breast and milk and stimulation of final digestion in the infant’s immature GI tract [66,67,68,69,70,71]. The neonate’s gastrointestinal (GI) system secretes insufficient stomach acid for degradation of proteins and has a limited enzymatic repertoire to digest and absorb amino acids, lipids, and sugars. Pre-digestion of these nutrients by mother’s breast and milk enzymes provides, therefore, a useful evolutionary service. The cleavage of nutrient molecules has three roles: producing nutrient molecules for infant energy needs, generating amino-acid building blocks for infant somatic and longitudinal growth, and providing immune defense mechanisms to defend the infant from pathogens.

Milk enzymes can be classified by their source: from maternal blood, through expression by mammary cells, or by immune cells, like polymorphonuclear neutrophils, in the breast [72]. Enzymes allowing for protein breakdown into peptides are peptidases and can be further categorized by their substrates as proteases breaking down proteins, lipases breaking down lipids, and others breaking down starches and other carbohydrates. The most active milk proteases, plasmin, trypsin, elastase, and cathepsin D (the last two expressed in milk), have the capacity to cleave more than 1100 unique pre-digested peptides from milk proteins [66,67]. The arrival of these pre-digested peptides triggers an increase in enzymatic digestion in the infant stomach. Among the most-actively pre-digested milk protein is β-casein, cleaved in the breast by cathepsin D, elastase, plasmin, proline endopeptidase and thrombin [66]. β-casein then releases short-chain bioactive peptides β-casomorphins (BCMs) encrypted within its molecule [73], which have also been found in cow’s milk, cheese and yoghurt. BCMs prolong the infant’s gastro-intestinal transit time, contributing to satiety and sleepiness, and also harbor anti-oxidative activity [73]. Hydrolysis of 42 milk proteins yielded 306 bioactive peptides, 28 of which had an anti-microbial action, 6 stimulated cell proliferation, and 10 inhibited ACE (angiotensin converting enzyme) [67].

Recent computational and mass-spectrometric techniques have allowed classification of milk enzymes on the basis of the number of cleavages they produce in the breast tissue and milk [71]. Two whey proteins, α-lactalbumin and lactoferrin, appear not to undergo any cleavage. Eight milk enzymes that take part in digestion of milk proteins, listed by the frequency of cleavages they perform, from 320 to 14, were as follows: the thrombolytic enzyme plasmin, proteolytic trypsin, cathepsin D, chymotrypsin, elastase, glutamyl endopeptidase-like enzyme, and proline peptidase. Milk enzymes were also classified by the number of proteins they subject to cleavage, from 24 to 5. The highest number of proteins are cleaved by cathepsin D [23] and the proline endopeptidase [17]. Plasmin, chymotrypsin, trypsin and elastase cleave 13 to 14, and pepsin and glutamyl endopeptidase cleave 10 and 5 milk proteins, respectively. Plasmin, trypsin, elastase, and cathepsin D cleave α_si_, β, and k caseins, The two proteins most affected by plasmin or trypsin digestion in the mammary gland were β-casein (89 cleavages), polymeric immunoglobulin receptor (32), and osteopontin (18 cleavages). Cathepsin D cleaved the same three proteins 78, 21, and 3 times, respectively, and elastase, the first two proteins 41 and 25 times, respectively.

The basic list of enzymes detected in infants’ organs has been known for at least three decades [69]. Among proteases, the infant stomach produces pepsins and chymosins capable of proteolysis and of curdling caseins, respectively. The infant intestine produces proteolytic entero-peptidases, and the pancreas produces proteolytic trypsins, chymoptypsins, elastases, and carboxypeptidases. Seven enzymes are concurrently present in human milk and neonatal infant stomach: plasmin, trypsin, cathepsin D, pepsin, elastase, chymotrypsin, and proline endopeptidase. Activities of plasmin, trypsin, and proline endopeptidase do not differ between milk and the infant stomach, but activities of milk-specific proteins cathepsin D, pepsin, elastase, chymotrypsin are approximately double in the infant stomach and display about ten times higher activity of the unique peptides associated with the four milk-specific proteins. Among carbohydrate-digesting enzymes, amylase is produced in salivary glands and the pancreas, while the infant intestine produces glucoamylase, sucrase-isomaltase, and lactase. The pH is neutral in the newborn infant’s stomach as little acid is released, and there is little gastric contractility [70]. Gastric pepsin is low at birth and increases in the course of infancy. As its concentration rises, pepsin hydrolyses the clot formed in the stomach between caseins and milk-fat globules.

In the newborn small intestine, the dominant lipases aiding fat digestion are pancreatic lipase-related protein 2 and bile-salt-stimulated lipase from the exocrine pancreas and the milk. The infant pancreas does not secrete sufficient pancreatic triglyceride lipase or phospholipase A_2_, and production of bile salts by infant liver is low [70]. Pancreatic lipase-related protein 2 is a dominant small-intestine lipase. On the other hand, gastric lipase is well developed in infants and is essential for infant digestion of triglycerides [70]. The infant duodenum and intestine produce four lipases, some associated with bile salts, and all are able to cleave phospholipids and triglycerides [69].

While lactose is secreted by mother’s breast, lactase is a homodimeric enzyme residing in the membranes of the small intestinal villus tips and is expressed mostly in the brush border of the mid jejunum [32]. Intestinal lactase activity is detectable in the fetal gut by eight weeks of gestation. Lactase activity is initially low, in contrast to the mature activities of sucrase, maltase, and isomaltase before birth, but it increases in parallel with gestational age. Under 34 weeks of gestation, lactase activity is at 30% of that at term. After the first breast milk intake, lactase activity rapidly increases and typically exceeds 98% efficiency in 5 days. At 10 days, lactase activity is greater in breast-fed than in IF-fed infants. Lactose that is not digested in the jejunum is fermented by bacteria in the terminal ileum and colon. During childhood, lactase levels and activity decrease from a peak at birth to less than 10% of preweaning level.

In about 65% of the global population, lactase expression declines after weaning, via epigenetic gene silencing, by 90 to 95%, leading to lactose intolerance. About 35% of the population are lactase persistent due to constitutive gene expression. In lactase non-persistent individuals, lactose-digesting colon bacteria can increase lactose processing in what is known as colonic adaptation [74].

#### 3.1.8. Milk and Infant Gastrointestinal Microbiota

The microbiome typically refers to the populations of bacteria residing in an infant’s or adult’s gut (mostly the intestine and colon). Adult women also have microbiota at other sites, such as the vagina, oro-nasal cavity, and skin. Pregnant women have additional microbiota in the breast [75], placenta and the amniotic fluid [76]. Adult gut microbiome consists of 3 × 10^14^ bacteria, more than 3 × 10^13^ cells in a typical adult’s body [77]. The beneficial functions of gut microbiome include metabolism of poorly digestible polysaccharides, such as HMOs, detoxifying toxic products, maintaining a protective barrier against pathogens, and supporting development of the host immune system. When antibiotics or other environmental factors cause dysbiosis of this system, the risk of metabolic diseases, such as diabetes, inflammatory and irritable bowel syndromes, obesity, allergy, autoimmune disease, and even some brain disorders, increases [77]. It is, therefore, helpful to understand the connection between the infant gut microbiome and human milk, as well as microbiome responses to several other influential factors. Colostrum, the first milk an infant drinks after birth, is particularly suitable for maximal stimulation of the infant’s gut microbiome. Colostrum has a 90:10 ratio of whey to casein and therefore provides the infant with ample quantities of αlactalbumin and lactoferrin, whose anti-bacterial protective functions are to prevent the adhesion of pathogenic bacteria *Helicobacter pylori*, *Eschericia coli*, *Klebsiella pneumoniae*, *Staphyllococcus aureus*, *S. epidermis*, and *Candida albicans* to the infant’s intestinal epithelial cells. They also play a role in infant brain, GI, and microbiome development [78]. A polyamine spermine, made in the breast tissue and also supplied also maternal diet, is a milk component that mediates formation of infant’s gut microbiota through maturation of GI epithelium and microbial proliferation. A number of HMOs, which are the third most abundant component of milk after lactose and lipids, support the anti-microbial lactic acid bacteria *Bifidobacterium*, *Lactobaccillus*, and *Bacteroides* spp. in the infant’s gut as they are able to metabolize HMOs, which are indigestible to infants [75,78]. Next, any amount of lactose that is not digested in the infant’s proximal small intestine becomes broken down in the distal jejunum and colon by the aforementioned lactic acid bacteria, which possess bacterial β-galactosidase activity [74]. Since this activity is low, additional lactose digestion and fermentation to lactate, short-chain fatty acids (SCFAs), and gases, such as H_2_, CO_2_, and CH_3_, takes place in the infant colon. The fermentation and SCFA production by lactic acid bacteria play an important role in the development of the infant immune system. SCFAs stimulate proliferation and differentiation of immune T cells, such as regulatory T and helper T cells. They also modulate immunoglobulin IgA and IgG secretion by immune B cells [77].

Milk can also influence the infant’s gut microbiome through its own bacterial population [75]. While milk was historically considered to be sterile, and any bacteria in it were considered a consequence of contamination from mother’s skin and the infant oral cavity, careful studies have shown that breast milk harbors bacteria and that the infant ingests these. Ingested Firmicutes bacteria, such as *Streptococcus* spp. and *Veilonella dispar*, are incorporated into the infant gut microbiome because they are also found in milk [79]. Milk, therefore, appears to have its own microbiome that contains commensal bacteria, and its function, in part, is to inoculate or seed the infant gut with them after birth [75]. At least six research groups have generated lists of up to more than 50 different types of milk bacteria, of which the most frequently present are species of *Staphylococcus*, *Streptococcus*, *Lactobacillus*, *Bifidobacterium*, and *Pseudomonas* [75]. In a detailed analysis of bacterial species found in human milk and the infant gut, *Staphylococcus* and *Streptococcus* were dominant in milk, with two and a half greater prevalence of *Streptococcus* over *Staphylococcus*, while *Bifidobacterium* and anaerobic *Bacteroides* dominated the infant gut, with *Bacterioides* slightly more numerous than *Bifidobacteria*. Bacteria in milk microbiome function as probiotics, which confer health benefits to infants, who consume between 8 × 10^4^ to 8 × 10^6^ commensal milk bacteria every day [76].

The types and diversity of breast milk bacteria are also influenced by maternal adiposity and quality of diet [26]. Obese mothers and maternal consumption of obesogenic high-fat, high cholesterol diets are associated with higher counts of *Staphylococci*, *Enterobacteriaceae*, and *Eschericia coli* and lower counts of *Bifidobacteria* and *Bacterioides* in the infant gut microbiome than is the case in normal-weight mothers, whose infant bacteriomes were similar to those in non-pregnant women [76].

There are several hypotheses on the route of bacterial entry into breast milk. One is that, during lactation, bacteria are transferred from the maternal gut via an entero-mammary pathway [75]. Special immune dendritic cells release bacteria from the gut lumen by penetrating the gut epithelium and by opening the tight junctions between intestinal epithelial cells. Then, the macrophages transport bacteria to mesenteric lymph nodes and to the blood connection with the mammary gland. The entero-mammary pathway operates also during pregnancy when the bacteria from the maternal gut microbiome, liberated by dendritic cells, enter the lymph and systemic circulation, and from there are transferred to the placenta, fetal membranes, and amniotic fluid [76]. During pregnancy, vaginal transmission of bacteria involves their ascent through the vagina to penetrate the decidua, fetal membranes and amniotic fluid. Evidence for the gestational bacterial transfer between mother and fetus was collected by sampling the meconium, the infant’s first bowel movement just before, or immediately after, the first breast-milk meal [76]. Using a fluorescent in situ hybridization technique, which precludes bacterial contamination, meconium microbiome was found to harbor 34 genera of Proteobacteria (featuring *Campylobacter*, *Escherichia*, and *Salmonella*), 36 genera of Firmicutes (featuring *Clostridium*, *Enterococcus*, *Lactobacillus*, *Staphylococcus*, *Streptococcus* and *Veillonella*), 8 genera of Bacterioidetes (including *Bacterioides*), 16 genera of Actinobacteria (featuring *Bifidobacterium* and *Micrococcus*), and 3 more genera of Fusobacteria and Tenericutes bacteria. All of these were derived from gut, vaginal, or amniotic fluid microbiomes.

The infant gut microbiome is also seeded with bacteria by other routes than by breast milk. One way is through birth by vaginal delivery, which supplies the infant’s gut microbiome with *Bacterioides*, *Bifidobacterium*, *Parabacterioides*, and *Escherichia*, while the fecal microbiota of infants delivered by cesarean section features bacteria from oral and skin microbiota and microbes from the surrounding environment. Transmission of vaginal microbes from mother at birth takes place in only some deliveries, and these bacteria are rapidly replaced by vaginal microbes delivered by breast milk [76]. Antibiotics cause disruption in the infant gut microbiome and shift it in favor of Proteobacteria (*Enterobacter*, *Escherichia*, *Klebsiella*, *Salmonella*) and drug-resistant bacteria, and away from Actinobacteria (*Actinomyces*, *Bifidobacterium*, *Micrococcus*). The use of antibiotics in early life increases the incidence of allergies, asthma, atopic disease, eczema, and type 1 diabetes [77]. With the introduction of solid food, the infant’s gut microbiota shift drastically from *Bifidobacterium*-dominant Actinobacteria to Bacterioidetes (*Bacterioides*, *Prevotella*) and Firmicutes (*Enterococcus*, *Lactobacillus*) bacterial genera. By age 3, the infants’ gut macrobiota are similar to those of adults [77]. 

### 3.2. Comparison of Infant Formula to Breast Milk

In 2016, only 38% or 1.5 million infants were breast fed globally, and the remaining 62% or 2.4 million relied on IFs for nutrition. In the United States, 75%, or 3 million infants, initiated breast feeding, but this declined to 67% or 2.7 million by the third month [31]. Industrial production and sales of infant formula generated 38.2 billion USD in 2021, and a few companies (Nestle, Danone, Abbott, Friesland Campina, and Heinz) controlled about 60% of the global market [80]. Besides the general acknowledgment [6,7,8] that breast milk is the ideal food for infants for at least 18 months, IF feeding for the vast majority of world newborns led to a global cost of production and overall environmental impact at 300 billion USD [81].

The purpose of IF feeding is not to closely duplicate human milk, which is qualitatively incomparable and highly complex, but to approximate the nutritional characteristics that human milk offers. In the US, the Food and Drug Administration does not proscribe a specific IF composition but approves manufacturer proposals and insures their adherence to acceptable ranges according to the Code of Federal Regulations Title 21 [62]. This code tracks concentrations of macronutrients, vitamins and minerals per 100 kilocalories of IF in whatever form it is prepared for consumption. Concentrations and doses listed are close to the breast milk values expressed in grams/100 mL milk shown in Table 1. In particular, milk protein, fat, and carbohydrate aim to conform to 1:2:4 proportions; polyunsaturated 18-carbon fatty acid with two unsaturated sites, like linoleic omega 6 acid, to 2–3% of total energy, and casein to whey ratio at 40:60. Minerals and vitamins are fortified as needed, osmolarity adjusted to 270 milliosmoles per liter to support infant’s kidney function, and bioactive ingredients, such as omega 3 and essential fatty acids, carnitine, taurine, polyamines, nucleotides, oligosaccharides, folates, prebiotics, and probiotics, are supplemented to approximate human milk, or to the results of studies indicating their benefits to infant growth and development [13].

In attempts to match human breast milk composition of 1% protein 3.8% fat, 7% lactose in 87 to 3388% of water, companies [13,31,80] use purified whey and casein from cow’s, or occasionally another domestic animal’s, milk for protein. With soy milk as the starter, fat is often provided by a blend of vegetable oils, vitamins, and minerals matched to human breast milk values. Probiotics and prebiotics isolated from fecal or food microbiota may also be substituted in IF milk.

#### Matching Infant Formula to Bioactivities of Breast Milk

The major unresolved issues and controversies are the extent to which IF captures the bioactivity of human breast milk. Several reviews compare the chemical composition of IF to breast milk and summarize the extent to which different versions of IF succeed, or still need improvement, in meeting this goal [13,41,78,80]. Notably, bioactive components need to be adjusted or substituted when low-fat cow milk is used to produce IF, as aspects of its composition differ from human milk [78]. Total protein content and casein to whey ratio have to be adjusted to 1% and a 40:60 ratio, respectively, because these two variables are about 3.5 and 7.5 higher in cow’s milk compared to breast milk (Table 1). This requires lowering the casein proportion of cow’s milk and increasing the proportion of whey proteins. Alpha-lactalbumin (αLA), an important whey protein, accounts for 28% of total human milk protein, and only 3% of cow’s milk protein [78]. αLA provides tryptophan, cysteine, lysine, and branched-chain amino acids (leucine, isoleucine, and valine) for infant nutrition, facilitates lactose synthesis and absorption of minerals, and blocks adhesion to the infant’s intestinal wall of the pathogen *Helicobacter pylori*. Supplementation of αLA to IF improves infant sleep (due to tryptophan) and immune system function (due to cysteine). αLA has antimicrobial action against *Escherichia coli*, *Klebsiella pneumoniae*, *Staphylococcus aureus*, *Staphylococcus epidermidis*, *Streptococci*, and *Candida albicans*. Other whey proteins, such as lactoferrin and lysozyme, are being tested for supplementation benefits.

As for immunoglobulins, human breast milk has secretory IgA, IgM, IgD, and IgE, while cow’s milk has mostly IgD, so secretory IgA (sIgA) is supplemented to cow’s milk-based IF. Taurine is the most abundant free amino acid in human milk at a concentration of 4.7 mg/100 kcal, and higher than in cow’s milk. It is an essential protein because it is obtained from the maternal diet. Taurine is required for perinatal central nervous system development and for neuroprotection. Synthetic taurine is mostly added to IF for premature infants. Folates are also bioactive molecules. Folic acid is an essential water-soluble B vitamin (B9) obtained from the maternal diet. Folates stimulate erythropoiesis in bone marrow. Deficiency causes congenital anomalies, neural tube defects during embryogenesis, deficient brain development, neurological diseases, and deficits in vitamin B12. Folate concentration in human breast milk is 12.3 μg/100 mL, and in cow’s milk only 5–10 μg/100 mL. Infant folate intake of 65 μg DFE (dietary folate equivalents)/day or 80 μg DFEs/day of folic acid are required for infants at age 6 to 12 months. All infant formulas must be supplemented to a minimum of 10 μg/100 mL and a maximum of 50 μg/100 mL folic acid.

Polyamines are organic molecules that contain more than two amino groups. Their important bioactivity entails interactions with DNA and RNA leading to synthesis, maintenance, and stability of nucleic acids. They can be imported through the maternal diet, but the breast makes high levels of polyamines, mainly spermine (173.4 nmol/dL) and spermidine (457.5 nmol/dL), with a lower amount of putrescine (82.4 nmol/dL). Polyamines are required for the formation of infant liver and pancreas, the immune system’s development, proliferation and maturation of the GI tract epithelium and the formation of intestinal microbiome. They prevent food allergies by decreasing mucosal permeability to antigenic proteins. However, their concentrations in breast milk are variable and differ from those in IF, so their supplementation is controversial.

Milk fat globule membrane (MFGM) is a bioactive organelle in breast milk secreted by the human alveolar epithelial cells and bound to lipolytic enzymes. It is composed mainly of lipids and proteins and their diameter at 1 month is 0.2 to 15 μm. Their nucleus consists of triglycerides, and their membrane has three layers constituted of phosphatidylcholine, sphingolipids, cholesterol, cerebrosides. and glycoproteins. MFGM produces bioactive molecules important in immune and gastrointestinal health, brain development, and cognitive function. Although substitution of MFGMs in IFs is considered desirable, it is absent from standard IFs when cow’s milkfat is replaced by vegetable oils, such as oleo, coconut, soy, palm, sunflower, and safflower oils, because their use in the manufacture of IFs with vegetable oils is not feasible. Bioactive fatty acids include some that humans can synthesize. These include saturated (SFAs) and MUFAs, the monounsaturated fatty acids. Humans cannot synthesize two 18-carbon fatty acids: PUFAs, the polyunsaturated fatty acids, like α-linolenic acid (ALA, 3 omega) and linoleic acid (LA, 6 omega) These are essential, as they have to be obtained from the diet and are precursors of long-chain PUFAs (LC-PUFAs). ALA is converted to EPA (20 carbons), and then to DHA (22 carbons, 3 omega), whereas LA is converted to AA (20 carbons, 6 omega). DHA and AA are transferred from mother to fetus in the third trimester through the placenta. Mean DHA and AA levels in breast milk worldwide are 0.32% and 0.47% of total fatty acids, respectively. The bioactive significance of DHA and AA is that they play a critical role in infant brain development, where they account for approximately 25% of the fatty acids in brain cells and in the retina. DHA constitutes 40–50% of PUFAs in the two organs. EPA contributes also to cardiovascular and immunological health. Since infants cannot synthesize LC-PUFAs, the enrichment of IF with DHA and ARA is desirable and has been shown to improve development of the visual system. Current supplementation of IF with fatty acids recommends a range of between 3 and 6 g/100 kcal. Allowable LA range is 300 to 1200 mg/100 kcal or 7 to 20% of total fatty acids, similar to the amount found in breast milk. When plant oils are used in IFs, supplementation with palm oils is necessary to increase the level of butyrate to that comparable to breast milk. LC-PUFA supplementation limits are optional in the USA at 2% of AA relative to total fatty acids, 1% for DHA (not to exceed the concentration of EPA), and DHA not to exceed 0.5%. In Europe, addition of DHA (20–50 mg/100 mL) is mandatory.

Finally, prebiotics and probiotics are also considered as bioactive molecules. The former are non-digestible HMO carbohydrates (di- oligo- and polysaccharides composed of glucose, fructose, galactose and other sugars), that reach the colon intact, where they are fermented by a specific group of bacteria. Almost 200 out of an estimated 1000 human HMOs have been found in breast milk. They decline in the course of lactation from 20.9–23.0 mg/mL to 7.0–12.9 mg/mL Their functions are to modulate beneficial intestinal bacteria *Bifidobacterium* and *Lactobacillus* and block pathogenic bacteria by acting as their competitive ligands on infant intestinal cells. HMOs are practically nonexistent in the cow’s milk. Production of HMOs is expensive and challenging, so IFs based on cow’s milk are supplemented with nondigestible carbohydrates, such as galacto-oligosaccharides (GOs) and/or fructo-oligosaccharides (FOs) and/or polydextrose (PDX), at approximate concentrations to human milk HMOs.

Probiotics are living bacteria that adjust intestinal microbial balance and resist intestinal colonization by pathogenic bacteria. Breast milk-fed intestinal flora is richer in beneficial and more varied bacteria than the flora of FI-fed infants. To approximate the two requires either supplementing IF milk with prebiotics or with cultures of bacteria and lactobacilli in order to mimic infant gastrointestinal colonization.

## 4. Discussion

The purpose of this review on the relevance of milk composition to human longitudinal growth from infancy through puberty is to present an overview of the relevant facts and examine controversies and areas open for additional research. The factual part of the review listed the composition of human milk and other milk options for infants from dairy animals, cow, camel, goat and sheep. This included the remarkable extent to which IF succeeds in matching human milk.

Literature sources on the effects of human milk composition frequently search for evidence for its causing early developmental programming of either longitudinal growth or obesity in later life. Testing this “origin hypothesis” involved seeking short-term correlations between adipokines or metabolic hormones and changes in aspects of infant growth, usually during the first year of life. Therefore, the developmental programming issue is the first area of controversy that requires both discussion regarding the duration of human development potentially vulnerable to nutritional variation, and additional research. The second area of controversy in need for additional research is the source of excess calories that may predispose infants toward a developmental trajectory of obesity in later life. These could reside in differences in quantities and qualities of breast milk and IF milk, exposure to hyperinsulinemia in the milk from insulin-resistant mothers, or consequences of post-deprivation catch-up growth. The third controversial and insufficiently pursued research area is the origin and role of probiotic bacteria in human milk and the infant gut microbiome, whose differential development may affect infant’s gut competence in digesting and absorbing milk nutrients. Advances in molecular and proteomic techniques now allow documentation of any lasting epigenetic changes during developmental programming. Epigenetic documentation may be the key approach to shedding light on the timing and control of developmental programming.

### 4.1. Critical Nutritional Periods for Developmental Programming of Longitudinal Growth

The first influential insight into the concept of critical neonatal nutritional periods was published about 65 years ago. Studies were performed on rats, mammals that give birth (like humans) to altricial or helpless young and that, unlike humans, display an early rapid growth rate that progressively and permanently declines at sexual maturity. The experimental manipulation used was that of altering neonatal milk availability by having a single dam suckle either two to three pups in small litters (SL) or fifteen or more pups in large litters (LL). These early studies provided four insights into nutritional critical periods. First, varying the abundance of milk dramatically affected infant growth rate from day 1 after birth and permanently changed pup growth trajectories. Body mass of SL pups was 2.4 times greater between the birth date and weaning week 3, 2.2 times greater by sixth week, and between 1.3 and 1.5 times greater at 3 and 6 months of measurement. At 6 months, when the growth rate dramatically declined, the large-litter (LL) rats weighed about 280 g, and SL ones up to 425 g [82,83,84]. The earliest postnatal period of fastest growth represented the critical period when overnutrition accelerated longitudinal growth, and undernutrition permanently reduced it. Undernutrition of SL pups by the same relative amount experienced by LL pups between weeks 3 and 6, 3 and 9, or 9 and 12, produced little or no effect on the rate of growth or the ultimate difference in longitudinal growth trajectory or body size [85].

The second insight was that diet-induced changes in growth rate and trajectory affected all organs in proportion to the change in total body mass. Kidneys, brain, heart, liver, and testes grew at different rates in SL and LL pups, and all were proportional to diet-induced changes in total body growth with three exceptions. Growth of the intestine was proportional to body mass in both groups until the weight of 100 g. Thereafter, growth of the intestine decreased significantly in LL rats to attain 6.2 g at their final weight of 280 g, while it increased to 8.6 g in SL rats at final study day 400 and a weight of 450 g. The second divergence was in accumulation of body fat which, between birth and the weight of 150 g, rose fourfold in SL pups, disproportionally above the body mass size. Beyond 150 g, the proportion of body fat in SL rats was adjusted and remained appropriate to body mass up to the 400 g final study measurement [82].

Another significant finding was that, between birth and a weight of 150 g, the pups were hyperphagic, eating 45 kcal/100 body mass. As the rate of longitudinal growth declined, rat food intake relative to body mass also declined, initially to 30 kcal, and later to 20 kcal/100 g of body mass [83]. This shows that a weight-appropriate control of feeding starts when the growth significantly declines or ceases, a marker of adult weight-maintenance stage. Ventromedial hypothalamic lesions of adult rats accelerated weight gain for a period of time. This was, again, accompanied by initial hyperphagic food intake during the fastest gain in size [83]. As was the case with neonatal rats, hyperphagia subsided as the growth rate declined or ceased.

The fourth, final, insight into the effect of the neonatal nutritional critical period is that it affects sexual maturation. Testes grew in SL pups disproportionately faster than in LL pups from birth up to 90 days of age, when their growth ceased in both groups. SL testes were 10% larger than LL testes [82]. The female rat vaginal opening typically occurs on the 42nd day of life, but opened at 30 days in SL pups at a weight of 70 g. In LL rats, this event occurred at 46 days and 65 g, thus showing that higher body mass triggered by differential neonatal nutrition affects sexual maturation programming by advancing the onset of puberty and is largely mediated by attained body mass [84].

These new insights into the role of differential neonatal nutrition on growth trajectories and changes in the onset of puberty were extended to the role of gestational/prenatal nutritional manipulation in another rat study [86]. Females were exposed to variable dietary exposure during pregnancy and pups during their postnatal life. Gestational undernutrition was achieved by feeding pregnant females 30% of ad libitum food, available during the entire pregnancy and lactation, while a control group had ad libitum access to food. After weaning, pups, in standardized litters of eight, were provided with either standard diet or a diet 23% higher in nutrient energy (4900 vs. 3500 kcal/kg). Maternal undernutrition during pregnancy and lactation resulted in fetal growth retardation. While litter size was unaffected, pups from undernourished dams weighed 3.9 g versus 6 g in ad libitum fed dams. Thus, dietary deprivation during pregnancy significantly reduced birth size. From parturition until weaning at day 22 of life, undernourished pups weighed significantly less (33.8 g) than the ad libitum-fed pups (52.5 g), suggesting that their growth trajectory was reduced. Caloric intake of pups was measured during the prepubertal period (PP, day 22 to puberty onset at day 40), post-pubertal period (PO, days 60 to 80), and mature adulthood (MA, days 100 to125 days). During the PP, undernourished rats provided with a hypercaloric diet ate 38% more kcal/g body weight per day than non-deprived pups eating a regular diet, while overeating by deprived pups on a regular diet and nondeprived rats on a hypercaloric diet was between 13 and 17% more. As they grew older, the stunted rats on a hypercaloric diet ate 77% more during the PO period and 230% more during the MA period while hyperphagia in the other two groups remained significantly lower. The remarkable results of the study suggested the extent to which intrauterine deprivation, by itself or combined with a hypercaloric diet, as well as just by itself, affected hyperphagia on the one hand and systolic blood pressure (BP_SYS_) and insulin and leptin blood concentrations on the other. By comparison with nondeprived offspring eating regular diet, BP_SYS_ increased by 9% with hypercaloric food, by 18% with prenatal undernutrition, and by 23% with the combination of the two. The same respective comparison for exposure to a hypercaloric diet by stunted rats revealed 50%, 75%, and 150% increases in plasma insulin concentrations, and 8%,10%, and 114% in plasma leptin concentrations. This study, thus, implicated gestational energy deprivation in lasting stunting of growth, persistent hyperphagia exaggerated by increased caloric density of food, and a remarkable association with increases in hypertension, hyperinsulinemia and hyperleptinemia.

What is the evidence for a nutritional critical period in humans? Compelling evidence for an early critical period in humans was collected during a Dutch famine of 1944 to 1945 during World War 2 (and other famines), with 43% maternal reduction in daily caloric intake during the first two trimesters of gestation [87,88,89], and possibly even at the time of conception [90]. Gestational energy deprivation increased risks for metabolic disruption, including obesity, type 2 diabetes [91], cardiovascular disease, and visual and auditory deficits throughout adult life [92]. Observed metabolic dysregulations were accompanied with epigenetic changes in histone methylation of particular metabolic genes, detected even 60 years after the fetal exposure to maternal starvation [90]. Other studies implicated hyperglycemia and hyperinsulinemia in the circulation during gestational diabetes in pregnant women leading to later-life obesity of their infants, despite large-to-gestational-age infant birth size [87]. Additional evidence attributes increased risk for coronary heart disease and diabetes to infants born small for gestational age [93]. In a study of 32 to 42-year old type-2 diabetics (700) and 3000 individuals with hypertension, there was a strong correlation between their small for gestational age birth size and their BMI between ages 3 and 11 years. Risk for diabetes and hypertension fell with each kg increase in birth size (odds ratios 0.67 and 1.2, respectively), while it increased with each kg decrease in birth size. This strongly suggests that small birth weight predicts large cumulative increase in the incidence of later cardiovascular disease and diabetes. The phenomenon is interpreted as a demonstration of a variety of adaptable phenotypic developmental pathways, including catch-up growth, which can compensate for early developmental deficit, but also produce metabolic dysfunction, with inadequate development of some tissues and organs.

Another line of evidence comes from surveys of global childhood longitudinal growth-stunting by up to 2 standard deviations (SDs) below the WHO Child Growth Standards [94]. The critical undernutrition exposure between 6 and 24 months after birth causes largely irreversible stunting. The process is compounded if the mother was also severely undernourished during pregnancy. In that case, infant length was usually 0.5 SD below the WHO standard at birth and declined to almost 2 SDs below the WHO standard by the end of the 2nd year [95]. While, in this situation, both weight for age Z score declined by half, and height for age Z score declined to 25% of the starting score, weight for length Z score remained relatively unchanged over 58 postnatal months [95], similar to the case of body organs adjusting in proportion to different rat longitudinal growth trajectories [82].

Humans are exceptional mammals in that they display a prolonged period of slow growth during childhood bracketed by the neonatal and pubertal periods of rapid growth. The fastest human growth rate of 2.5 cm/week is during the gestational weeks 20 and 24, mostly mediated by nutrient-induced IGF-1 and several other growth factors [96]. That is why maternal starvation during this period is so metabolically and physically damaging [88,91,92]. This early rapid growth rate is 52 times faster than during year 1 of life, when infants grow by 25 cm/year. During the second year, growth declines by half to about 12 to 13 cm/year. Between the second and eighth year, height velocity of both girls and boys is usually only 6 to 8 cm/year with a transient increase due to the onset of adrenarche (increased production of androgens by the adrenal gland) at that time. During childhood, growth hormone (GH) assumes a key role in the control of linear growth [62] and is stimulated by insulin, leptin and assisted by thyroid T4 hormone. GH stimulates the release of IGF-1 from the liver. Then, between ages 10 and 11 in girls and 12 and 13 in boys, pubertal growth spurt occurs with an average peak height velocity (PHV) of about 8 to 10 cm/year in girls and 10 to 12 cm/year in boys. This is achieved through activation of the hypothalamic–pituitary–adrenal axis initiated by increased pulsatility of the gonadotrophic hormones which stimulates secretion of sex hormones estradiol and testosterone. The site where both circulating and local hormones achieve linear growth is the epiphyseal growth zone (EGZ) at the ends of the long bones. Elongation of long bones responds both to circulating hormones and to direct local activation of chondrocyte growth by GH, IGF-1, leptin, and sex steroids. After PHV, the growth rate steeply declines as females attain menarche and boys complete gonadarche and, over the next 2 to 3 years, epiphyseal growth zones in long bones close in response to high titers of estradiol.

As was the case for overnourished and undernourished rat pups, there is evidence that human sexual maturation can be affected by variation in neonatal nutrition. Precocious onset or rapid progression of adrenarche and puberty have been associated with being born small-for-gestational-age or preterm, with accelerated childhood linear growth and weight gain, with catch-up growth, as well as with childhood obesity. Similarly, female infants displaying accelerated linear growth between birth and childhood ages of 6 to 9 and 9 to 11 years, whether they had normal-for-gestational-age or small-for-gestational-age birth weight, display earlier adrenarche and onset of puberty [97]. In addition, obesity per se, without any apparent nutritional manipulation, is associated with premature onset of puberty in girls, but not in boys. Therefore, similar to the effects of early nutritional manipulation on vaginal opening in female rats [84], obesity in girls should be considered an independent risk factor for precocious puberty for premature closure of epiphyseal growth zones and cessation of longitudinal growth [98].

As the critical period of neonatal nutritional vulnerability in rats occurred during the period of fastest longitudinal growth and, besides the body-size trajectory, affected also the timing of sexual maturation at the end of this growth period, the existence of possible additional nutritional critical periods in humans could be associated with growth-rate transitions during the long duration from conception to the end of puberty. Therefore, to resolve uncertainties about possible additional critical periods in human longitudinal growth, it is important and desirable to extend the period of study of nutritional influence over longitudinal growth to the entire period of human growth from conception through the end of the pubertal growth spurt, rather than confining it to the early postnatal period. As the epigenetic gene markers persist into adulthood for up to 60 years [90], they could be used to identify any nutritional effects on successive stages of childhood and adolescent growth to identify any connections to possible metabolic dysfunctions.

### 4.2. Early Risk Factors for Developmental Programming for Obesity in Later Life

A concern for inadvertent early programing for obesity in later life is possibly more important than preoccupation with early nutrition and longitudinal growth. The reasons are the rising prevalence of obesity during childhood and adolescence in the US and developed countries in general, and the great difficulty in reducing obesity once it is established.

From 1975 to 2016, the global prevalence of obesity in children and adolescents aged 5 to 19 years increased from 0.7% to 5.6% for girls and from 0.9% to 7.8% for boys [99]. Obesity during childhood usually continues into adulthood and is associated with cardiometabolic pathologies of hypertension, coronary heart disease, type 2 diabetes, and psychosocial comorbidity, as well as premature adult mortality. The development and perpetuation of obesity is largely explained by a bio-socio-ecological framework, whereby biological predisposition by different ethnicity, socio-economic circumstances driving consumption of high-density, ultra-processed, often inexpensive foods in low-income settings, and environmental factors, such as reduced opportunities for physical activity and access to healthy food, interact to promote deposition and proliferation of adipose tissue. Once obesity is established, efforts at reducing weight, whether they are attempted via behavioral means, such as dieting [100], or pharmacological means, such as injections of incretin-receptor agonists semaglutide [101] and tirzepatide [102], lead to weight-regain and relapse to pre-intervention weight within weeks, months, or years [100,103].

An explanation of obesity relapses after deliberate weight loss has recently been revealed as representing an epigenetic obesity “memory” in the adipose tissue [104]. This remarkable study first compared gene expression in the subcutaneous and omental adipocytes of obese individuals with a BMI of 48 kg/m^2^ and healthy-weight controls with a BMI of 24 kg/m^2^. After a 25% reduction in BMI in the obese group, adipocytes and their progenitor cells displayed downregulation of genes controlling adipose metabolism and function and upregulation of fibrosis and inflammatory pathways. This was followed by a study inducing obesity in young mice by feeding them a high-fat diet (HFD). Similar epigenetic changes were induced by obesity but also persisted after restoration of pre-obesity weight. The metabolic disruptions that developed during the obese state, such as hyperglycemia and hyperinsulinemia, were only partially corrected by weight loss, and remained more expressed after 48-days rather than 24-day duration of obesity. Molecular analyses revealed that the memory of obesity in adipocytes involved changes in the expression of promoters and enhancers of several metabolic genes. When presented again with the HFD, weight gain and obesity were reinstated more rapidly than they had developed originally. In this study, obesity produced stable epigenetic changes in the adipose tissue which led to rapid obesity rebound to obesogenic stimuli and provided a plausible mechanism for this intractable health problem.

Among possible developmental origins of later-life obesity are uneven caloric intake during the neonatal period, extended exposure to hyperinsulinemia either in utero or in maternal milk, and differences in birth weight. An additional candidate discussed in Section 4.3 is a dysbiosis of the infant gut metabolome controlling the infant’s capacity to digest and absorb nutrients.

#### 4.2.1. Uneven Infant Caloric Intake as a Source of Later-Life Obesity

The focus of many studies on the nutritional neonatal programming of obesity or longitudinal growth have focused on adipokines and metabolic hormones in the breast milk and its composition (Section 3.1.6), but remarkably few have quantified caloric intake of breast-fed and IF-fed infants [105,106]. Breast milk is delivered on demand when infant wants to suck, and the quality of milk changes during individual feeding episodes, from higher protein and fructose concentration at the start, to an increase in fat content toward the end, of the feeding bout and of the phase of lactation [28]. On the other hand, IF composition of protein, fructose, and fat does not change (Section 3.2) and therefore produces different caloric delivery of the three nutrients as a function of time, compared to breast milk composition that varies during individual feeding bouts. In addition, IF milk delivery is to an important extent under maternal control, where its provision may inappropriately respond to infant fussing for reasons other than hunger. Measurement of caloric intake of infants has been studied infrequently as it is inconvenient to measure changes in infant weight after individual feedings, as well as changes in the major milk nutrients of different caloric density during feeding bouts, while there may be a difference in the caloric intakes of breast-fed and IF-fed infants

In addition, many studies of hormonal influences in developmental programming may have been misguided by controversial interpretations of how hormones control human appetite. The prevailing hypothesis is that hormones ghrelin and insulin and adipokines leptin and adiponectin directly guide adult hunger and satiation and therefore should also influence infant appetite. The controversy revolves around data interpreting a coincidence between hunger at the onset of a meal and blood concentrations of ghrelin [107] as evidence of their causal relationship, and the suppression of hunger by leptin injections in individuals unable to secrete it [52,53] as evidence of its control of meal-associated satiation. This point of view ignores the contradictory evidence that, of the usual four gut peptides whose actions are attributed to either hunger (ghrelin) or satiation (cholecystokinin-CCK, glucagon-like peptide 1-GLP-1, and peptide YY-PYY, GLP-1, PYY, ghrelin, and CCK), and ratings for hunger, none of them appear to control either. The secretion of all of these simply follows the time course of nutrient transit through the GI tract and is unaffected by variations in energy availability during a meal that either preceded, or followed, energy expenditure from exercise [108]. In addition, attributing a direct role to leptin and insulin in the control of appetite is contradicted by findings that human appetite responds to these messengers only when nutrients are taken by mouth and processed by the GI tract [109]. If they are introduced parenterally, that is by intravenous injection, they have no correlation with either hunger or satiation. Instead, leptin increases hunger when its concentration declines with energy deprivation and its inhibitory control of hypothalamic orexigenic centers wanes [54]. The argument that the control of meal-to-meal intake in adults is controlled and coordinated by gastrointestinal signals is supported by substantial evidence [110]. Only the analogs of the incretin GLP-1 have been shown to suppress hunger [111] and cause weight loss, as shown by the commercial success of GLP-1 receptor agonists semaglutide and tirzepatide. Even this appetite-suppressing effect of GLP-1 analogs may be indirect, as GLP-1 secretion [112], together with that of PYY [113], suppresses gastric emptying when unabsorbed nutrients from large meals reach the ileum and colon, an effect called the ileal brake. The relevant point is that the effect is produced by a gut hormone (GLP-1) involved in GI processing of eaten food.

#### 4.2.2. The Role of Infant Birth Weight in Programming of Later-Life Obesity

Obese women between 20 and 40 years of age usually give birth to large-for-gestational age infants (LGA), and their proportion in 2012 was 32%. This is true for all three categories of obesity: class I BMI 30 to 34.9 kg/m^2^, class II BMI 35 to 39.9 kg/m^2^, and class III BMI > 40 kg/m^2^. Class III obese women delivered infants weighing between 3.3 and 3.4 kg [114]. There is a robust positive association between birth weight and BMI in later life [115]. BMI at age 20 to 26 had increased 0.5 to 0.7 kg/m^2^ for each 1 kg increment in birth weight. Since there is a positive association between infant birth size and maternal as well as paternal birth weights, genetic, intergenerational, or environmental influences may also be at play.

Among the possible causes of LGA birth sizes is obesity-associated maternal insulin resistance. Obese mothers display carbohydrate intolerance and associated hyperglycemia and hyperinsulinemia in their circulation [61], as do type 2 diabetic mothers and women with gestational diabetes [115]. During pregnancy, maternal hyperglycemia stimulates the developing fetal pancreas to increase insulin secretion, because maternal insulin does not cross the placenta. This insulin can be measured in amniotic fluid. At birth, some LGA infants are already obese. After birth, neonatal exposure to high insulin concentrations is attributed to both maternal hyperglycemia and hyperinsulinemia in the breast milk. Prolonged infant exposure to hyperglycemia and hyperinsulinemia leads to late life obesity, which can be correlated with insulin concentration in amniotic fluid. High insulin concentrations in the milk of insulin-resistant obese or diabetic mothers, compounds infant exposure to insulin, which rules overall fat synthesis [56], evident in subcutaneous sites of insulin injections [116]. At age 14 to 17 years, offspring of mothers with gestational diabetes had a BMI of 26 kg/m^2^ compared to 20.9 kg/m^2^ in control subjects (115). In the US *Growing Up Today* study, a 1 kg-increment in birthweight among full-term infants was associated with a 30% risk of over-weight at ages 9 to 40 years (115). At this time, it is not clear whether persistence of obesity and hyperinsulinemia in later life is caused by epigenetic changes in the pancreatic cell function or central changes in neural control of appetite.

Paradoxically, infants born small for gestational age (SGA) also develop obesity in later life. SGA birth size predisposes infants to development of abdominal obesity (115), type 2 diabetes, hypertension, hyperlipidemia [117], reduced circulatory capacity and hypertension [118], and smaller head circumference and reduced cognitive capacity [119]. Central or truncal obesity, measured by the waist-to-hip (WH) ratio or the subscapular-to-triceps skinfold ratio, predicts cardiovascular risk [115]. Central obesity is associated with hypertension, dyslipidemia, glucose intolerance and diabetes, and increased risk of ischemic cardiovascular disease, a cluster of symptoms called metabolic syndrome. The risk of these symptoms is expressed as percentage change in risk estimate for each kg increase in birth weight. These estimate changes are −5 to −6% for skinfold ratios, −0.8% to −1.2% for WH ratios, −14% for HOMA-IR measure of insulin resistance, and odds ratios (ORs) of 0.5 to 0.6 for reducing dyslipidemia, carbohydrate intolerance, hypertension, insulin resistance and skinfold thickness. Possible agents for developing SGA birth weight are maternal low-protein intake during pregnancy, increases in cortisol concentrations, changes in secretion of GH and IGF-1, and even changes in sympathetic and brain control of growth (115). The current hypothesis is that the mechanisms leading to later-life obesity are different in LGA and SGA births. LGA leads to eventual obesity and associated comorbidities and may be associated with maternal hyperglycemia amd hyperinsulinemia, and in LGA birthweight is inversely associated with central obesity, where body fat is preserved at the expense of lean mass.

Thus, the quest for an understanding of early developmental programming of infant longitudinal growth and propensity for obesity is at this point unsettled and controversial. Therefore, the second recommendation for additional research is for studies of infant caloric intake or energy supply providing different fetal or postnatal energy support to determine circumstances for any possible epigenetic obesity programming though the entire period of human growth. from conception through the end of puberty.

### 4.3. Role of Gut Microbiome in Developmental Programming for Growth or Obsity

A remarkable development in the past five or so years has been the appearance of research on the role of the maternal and infant gut microbiome in the control of infant’s longitudinal as well as ponderal growth. This area of research may have escaped scrutiny because of the relative recency of information on gut microbiome effects on human health, where the number of publications in the past ten years rose from 774 to 14,205. Recent studies attribute an important role in the control of longitudinal growth to programmed maturation of the infant gut microbiome during infancy and childhood, as this determines GI capacity to assimilate nutrients necessary for infant growth and development. Four recent studies provide an overview of the development and maturation of the infant gut microbiome from its start during pregnancy to the age of 2 years [120]. Another identifies bacteria associated with the speed of linear ponderal growth during the infant’s first 2 to 6 months [121]. The third compares the involvement of infant gut bacteria during growth stunting or early undernutrition, as opposed to their facilitation of healthy longitudinal growth [122]. The fourth describes the epigenetic programming of greater weight-to-length Z score after infections with cytomegalovirus [123]. The point is made in all four studies that the gestational and neonatal microbiome period represents a critical window in the infant developmental program, defined by interconnections between metabolic, endocrine, neural, and immune processes, in addition to environmental constraints.

The intrauterine environment of healthy urban women is characterized by a distinct microbiome typically dominated by one of four *Lactobacillus* bacteria [120]. Preterm birth is associated with reduced diversity of maternal gut microbiota and lower abundance of *Bifidobacterium*, *Streptococcus*, and *Clostridium*. The abundance of *Actinomyces naeslundii* in maternal saliva is negatively associated, and *Lactobacillus casei* positively associated, with birth weight. When maternal undernutrition compromises infant growth and contributes to fetal stunting and small for gestational age (SGA) birth, prevalence of maternal vaginal *Lactobacilli* is reduced and replaced by increased abundance of *Prevotella*, *Gemella*, and *Cynobacterium*. As for fetal microbiota, lower newborn length-to-age z score was associated with the presence of *Sneathia sanguinengens* and *Peptostreptococcus anaerobius* in both maternal vagina and placenta, while smaller newborn head circumference was observed with the presence of *Phaseolactobacterium succinatutens* and *Lachnospiraceae bacterium*. In the preterm gut microbiota, an early facultative population of anaerobic *Bacilli* is followed by obligate anaerobes *Bifidobacterium*, *Bacterioides*, *Clostridium* and *Gammaproteobacteria.* These provide fermentation-based metabolism, defining an immature stage of the infant gut microbiome. These bacteria are also able to utilize HMOs in breast-fed infants, which increases infant capacity to harvest nutrient energy.

As infant gut microbiome matures, the bacteria most contributing to length-for-age z score within the first 6 months of infant’s life are *Streptococcus thermofilus* and *Bifidobacterium longum*. Maternal milk bacteria enhancing longitudinal growth at this stage of development include in the genus Pseudomonas *Protobacterium*, *Staphylococcus*, and *Streptococcus*. Intrauterine administration of the antibiotic penicillin was positively associated with infant ponderal growth between 2 and 6 months of age and was accompanied in the infant gut by an abundance of orders Proteobacteria, Bacterioides, and Firmicutes. Actinobacteria, Firmicutes, and Bifidobacteriales were positively associated with infant weight and BMI growth speed during the same postnatal age [121].

In a study comparing the maturation of healthy and challenged infant gut microbiomes [122], bacterial profiles and their enzymatic activities were described as facilitating both longitudinal and ponderal growth. The actinobacterium *Bifidobacterium longum* was a predominant infant gut bacterium up to 12 months of age. Four other species, *B. breve*, *B. bifidum*, *B. pseudocatelunatum*, and *B. kashiwanohense* were abundant during early months, before being outnumbered by *Faecalibacterium* (a Firmicute) and *Prevotella* (a Bacteroidete) between 12 and 18 months. During the first two months, the bacterial metabolism in the infant microbiome that predicted longitudinal growth and the velocity of length-for-age growth between 3 and 6 months entailed the support of several biosynthetic metabolic pathways: [1] for purine and pyrimidine nucleotides, [2] for B vitamins (flavin, folate, biotin, thiazole, and cobalamin), and [3] for amino acid and FA synthesis. Between 3 and 6 months, bacterial metabolic pathways in the infant gut microbiome that supported longitudinal growth and growth velocity also encoded fermentation and carbohydrate biosynthesis, while carbohydrate and amino acid degradation pathways were predictive of growth after 6 months of life. Bacteria that were predictive of ponderal, rather than longitudinal, growth were *Escherichia coli*, *Bacterioides fragilis*, and *Megasphaera micronuciformis*, although some of these supported both weight-for-age and length-for-age z scores.

Infants exposed to acute serious malnutrition or maternal HIV infection had a disturbed gut microbiota, which featured a depletion of *Bifidobacteria*, reduced amino acid biosynthesis, impaired growth and fetal stunting featuring *Faecobacterium prausnitzii* [122]. Maternal infection with human cytomegalovirus, transmits this herpes virus to breast milk, from where it transfers to the infant microbiome [123]. The infection decreases the abundance of Bifidobacteria in infant gut and stimulates weight growth at the expense of longitudinal growth at one month, but not at 6 months.

The introduction of solid food after the infant’s second year leads to structural and functional diversity in the infant gut microbiota in the direction of the adult state. It becomes dominated by species capable of digesting glycans, mucin, and complex carbohydrates. SCFAs and microbial fermentation produced by infant bacteria at an early age have also been linked to IGF-1 and somatotropic facilitation of longitudinal growth. It is speculated that changes in the infant gut microbiome during growth-stunting acute starvation may be transmitted intergenerationally by way of epigenetic changes as undernourished mothers of short stature tend to give birth to small-for-gestational age infants [120].

The above evidence indicates the important role of the composition, maturational metabolism, and nutritional vulnerability of infant gut microbiota during the infant’s developmental longitudinal and ponderal growth program. These data support the third recommendation for exploration of the relevance of factors influencing the infant gut microbiome in the developmental programming toward late-life statural growth or obesity, from conception through the end of pubertal growth.

## 5. Conclusions

This review explored the facts and controversies regarding the extent to which the milk of several dairy animals and IF approximate special properties and bioactivities of breast milk. It provides evidence that early exposure to undernutrition during the very rapid fetal and early infancy growth predominantly and permanently stunts longitudinal growth trajectory in both animals and humans, and is often followed in later life by obesity, metabolic dysfunction, and sometimes by precocious sexual maturation. A knowledge gap needs to be addressed as to whether there may be additional critical periods in human development which is characterized by a relatively long period of slow childhood growth bracketed by the rapid fetal and prepubertal growth spurts. This also raises attention to possible quantitative differences in caloric intake of breast-fed and IF-fed infants that may influence their developmental fatness programming. Finally, a knowledge gap needs to be addressed regarding the role of infant microbiome composition and development in the possible epigenetic programming for longitudinal growth or fatness in later life. Three recommendations are proffered: (1) that the research be extended to encompass the entire period of human growth from conception to the end of puberty; (2) to measure possible differences in nutrient energy intake or energy availability at different stages of growth as possible epigenetic programming influencing later-life obesity; and (3) to address the role of quality and the development of the infant gut microbiome for a better understanding of the interaction between critical nutritional periods and the control of human longitudinal growth and later-life obesity.

## References

[1] Oftedal O.T., Ogra P.L., Walker W.A., Lőnnerdal B. (2020). The evolution of lactation in mammalian species. Milk, Mucosal Immunity and the Microbiome: Impact on the Neonate.

[2] McNeilly A.S., Robinson I.C., Houston M.J., Howie P.W. (1983). Release of oxytocin and prolactin in response to suckling. Br. Med. J. (Clin. Res. Ed.).

[3] Valtcheva S.A., Issa H., Bair-Marshall C.J., Martin K.A., Jung K., Zhang Y., Kwon H.-B., Froemke R.C. (2023). Neural circuitry for maternal oxytocin release induced by infant cries. Nature.

[4] Su J., Su Y. (2018). A touch-scaffolded model of human prosociality. Neurosci. Biobeh. Rev..

[5] McNeilly A.S., Tay C.C., Glasier A. (1994). Physiological mechanism underlying lactational amenorrhea. Am. N. Y. Acad. Sci..

[6] Office of the Surgeon General (US), Centers for Disease Control and Prevention (US), Office on Women’s Health (US) (2011). The Surgeon General’s Call to Action to Support Breastfeeding. Publications and Reports of the Surgeon General.

[7] Gartner L.M., Morton J., Lawrence R.A., Naylor A.J., O’Hare D., Schanler R.J., Eidelman A.I. (2005). American Academy of Pediatrics Section on Breastfeeding. Breastfeeding and the use of human milk. Pediatrics.

[8] WHO Infant and Young Child Nutrition—Global Strategy on Infant and Young Child Feeding. Report by Secretariat. http://apps.who.int/gb/archive/pdf_files/WHA55/ea5515.pdf.

[9] MacHugh D.E., Larson G., Orlando L. (2017). Taming the past: Ancient DNA and the study of animal domestication. Annu. Rev. Anim. Biosci..

[10] Osthoff G. (2011). Milks of non-dairy animals. Encyclopedia of Dairy Sciences.

[11] Yi D.Y., Kim S.Y. (2021). Human breast composition and function in human health: From nutritional components to microbiome and microRNAs. Nutrients.

[12] Cerasani J., Ceroni F., De Cosmi V., Mazzocchi A., Morniroli D., Rogero P., Mosca F., Agostoni C., Gianni M. (2020). Human milk feeding and preterm infants’ growth and body compositions: A literature review. Nutrients.

[13] Guo M. (2024). Human milk biochemistry and infant formula manufacturing technology (Chapter 10). Functional Foods.

[14] Almasri R.S., Bedir A.S., Ranneh Y.K., El-Tarabily K.A., Al Raish S.M. (2024). Benefits of Camel Milk over Cow and Goat Milk for Infant and Adult Health in Fighting Chronic Diseases: A Review. Nutrients.

[15] Arain M.A., Salman H.M., Ali M., Khaskheli G.B., Barham G.S., Marghazani I.B., Ahmad S. (2024). A review of camel milk composition, techno-functional properties and processing constraints. Food Sci. Anim. Resour..

[16] Faye B., Bengoumi M., Al-Masaud A., Konuspayeva G. (2015). Comparative milk and serum cholesterol content in dairy cow and camel. J. King Saud Univ. Sci..

[17] Gorban A.M., Izzeldin O.M. (2001). Fatty acids and lipids of camel milk and colostrum. Int. J. Food Sci. Nutr..

[18] Konuspayeva G., Faye B., Loiseau G. (2009). The composition of camel milk: A meta-analysis of the literature data. J. Food Comp. Anal..

[19] Cimino F., Catapano A., Villano I., Di Maio G., Petrella L., Traina G., Pizzella A., Tudisco R., Cavaliere G. (2023). Invited review: Human, cow, and donkey milk comparison: Focus on metabolic effects. J. Dairy. Sci..

[20] Li L., Liu X., Guo H. (2018). The nutritional ingredients and antioxidant activity of donkey milk and donkey milk powder. Food Sci. Biotechnol..

[21] Stergiadis S., Nørskov N.P., Purup S., Givens I., Lee M.R.F. (2019). Comparative nutrient profiling of retail goat and cow milk. Nutrients.

[22] Turkmen N. (2017). The nutritional value and health benefits of goat milk components. Nutrients in Dairy and Their Implications for Health and Disease.

[23] Miller E.M. (2017). What is significant about a single nursing session? An exploratory study. Am. J. Hum. Biol..

[24] Andreas N.J., Kampmann B., Mehring Le-Dore K. (2015). Human breast milk: A review on its composition and bioactivity. Early Hum. Dev..

[25] Fujita M., Roth F., Lo Y.J., Hurst C., Vollner J., Kendell A. (2012). In poor families, mother’s milk is richer for daughters than sons: A test of Trivers-Willard hypothesis in agropastoral settlements in Northern Kenya. Am. J. Phys. Anthropol..

[26] Garcia-Mantrana I., Bertua B., Martinez-Costa C., Collado M.C. (2016). Perinatal nutrition: How to take care of the gut microbiota?. Clin. Nutr. Exp..

[27] Jenness R. (1979). The composition of human milk. Semin. Perinatol..

[28] Hytten P.E. (1954). Clinical and chemical studies in human lactation. Br. Med. J..

[29] Insull W., Hirsch J., James T., Ahrens E.H. (1959). The fatty acids of human milk. II. Alterations produced by manipulations of caloric balance and exchange of dietary fats. J. Clin. Investig..

[30] Castellote C., Casillas R., Ramírez-Santana C., Pérez-Cano F.J., Castell M., Moretones M.G., López-Sabater M.C., Franch À. (2011). Premature delivery influences the immunological composition of colostrum and transitional and mature human milk. J. Nutr..

[31] Martin C.R., Pei-Ra Ling Blackburn G.L. (2016). Review of infant feeding: Key features of breast milk and infant formula. Nutrients.

[32] Romero-Velarde E., Delgado-Franco D., García-Gutiérrez M., Gurrola-Díaz C., Larrosa-Haro A., Montijo-Barrios E., Muskiet F.A.J., Vargas-Guerrero B., Geurts J. (2019). The importance of lactose in the human diet: Outcomes of a Mexican consensus meeting. Nutrients.

[33] Shkembi B., Huppertz T. (2023). Glycemic responses of milk and plant-based drinks: Food matriz effects. Foods.

[34] Rosenstein D., Oster H. (1988). Differential facial responses to four basic tastes in newborns. Child. Dev..

[35] Forestall C.A. (2017). Flavor perception and preference development in human infants. Ann. Nutr. Metab..

[36] Delaveau P. (2004). L’obésité: Une épidémie á maitriser. Remarques sur l’alimentation. Ann. Pharm. Fr..

[37] Tishkoff S.A., Reed F.A., Ranciaro A., Voight B.F., Babbitt C.C.S., Silverman J.S., Powell K., Mortensen H.M., Hirbo J.B., Osman M. (2007). Convergent adaptation of human lactase persistence in Africa and Europe. Nat. Genet..

[38] Durá-Travé T., Gallinas-Victoriano F. (2023). Pregnancy, breastfeeding, and vitamin D. Int. J. Mol. Sci..

[39] Hermesch A.C., Kernberg A.S., Layoun V.R., Caughey A.B. (2024). Oxytocin: Physiology, pharmacology, and clinical application for labor management. Am. J. Obstet. Gynecol..

[40] Sala N.L., Luther E.C., Arballo J.C., Cordero Funes J.C. (1974). Oxytocin reproducing reflex milk ejection in lactating women. J. Appl. Physiol..

[41] Insel T.R. (1992). Oxytocin—A neuropeptide for affiliation: Evidence from behavioral, receptor autoradiographic, and comparative studies. Psychoneuroendocrinology.

[42] Freund-Mercier M.J. (2022). How oxytocin became overtime the attachment-mediating hormone. Biol. Aujourdhui..

[43] Çatlı G., Olgaç Dündar N., Dündar B.N. (2014). Adipokines in breast milk. J. Clin. Res. Pediatr. Endocrinol..

[44] Doneray H., Tavlas G., Ozden A., Ozturk N. (2023). The role of breast milk beta-endorphin and relaxin-2 on infant colic. Pediatr. Res..

[45] Häusler S., Lanzinger E., Sams E., Fazelnia C., Allmer K., Binder C., Reiter R.J., Felder T.K. (2024). Melatonin in Human Breast Milk and Its Potential Role in Circadian Entrainment: A Nod towards Chrononutrition?. Nutrients.

[46] Kon I.Y., Shilina N.M., Gmoshinskaya M.V., Ivanushkina T.A. (2014). The study of breast milk IGF-1, leptin. Ghrelin and adiponectin levels as possible reasons for high weight gain in breast-fed infants. Ann. Nutr. Metab..

[47] Kratzsch J., Bae Y.J., Kiess W. (2018). Adipokines in human breast milk. Best. Pract. Res. Clin. Endocrinol. Metab..

[48] Lu M., Xiao H., Li K., Jiang J., Wu K., Li D. (2017). Concentrations of estrogen and progesterone in breast milk and their relationship with the mother’s diet. Food Funct..

[49] Mizuta H., Amino N., Ichihara K., Harada T., Nose O., Tanizawa O., Miyai K. (1983). Thyroid hormones in human milk and their influence on thyroid function of breast-fed babies. Pediatr. Res..

[50] Mól N., Tomasik P., Klimasz K., Zasada M., Kwinta P. (2022). Irisin concentration in infant formulas and breast milk. Minerva Pediatr..

[51] Toorop A.A., van der Voorn B., Hollanders J.J., Dijkstra L.R., Dolman K.M., Heijboer A.C., Rotteveel J., Honig A., Finken M.J.J. (2020). Diurnal rhythmicity in breast-milk glucocorticoids, and infant behavior and sleep at age 3 months. Endocrine.

[52] Farooqi I.S. (2008). Monogenic human obesity. Front. Horm. Res..

[53] Farooqi S., O’Rahilly S. (2006). Genetics of obesity in humans. Endocr. Rev..

[54] Flak J.N., Myers M.G. (2016). Minireview: CNS mechanisms of leptin action. Mol. Endocrinol..

[55] Perakakis N., Farr O.M., Mantzoros C.S. (2021). Leptin in leanness and obesity: JACC State-of-the art review. J. Am. Coll. Cardiol..

[56] Norton L., Shannon C., Gastaldelli A., DeFronzo R.A. (2022). Insulin: The master regulator of glucose metabolism. Metabolism.

[57] Borer K.T. (2014). Counterregulation of insulin by leptin as key component of autonomic regulation of body weight. World J. Diabetes.

[58] Sinkiewicz-Darol E., Adamczyk I., Łubiech K., Pilarska G., Twaružek M. (2022). Leptin in human milk—One of the key regulators of nutritional programming. Molecules.

[59] Palou M., Picó C., Palou A. (2018). Leptin as a breast milk component for the prevention of obesity. Nutr. Rev..

[60] Mazzocchi A., Gianni M.L., Morniroli D., Leone L., Roggero P., Agostoni C., De Cosmi V., Mosca F. (2019). Hormones in breast milk and effect on infant’s growth. Nutrients.

[61] Dadres G.S., Whitaker K.M., Haapala J.L., Foster L., Smith K.D., Teague A.M., Jacobs D.R., Kharbanda E.O., McGovern P.M., Schoenfuss T.C. (2019). Relationship of maternal weight status before, during, and after pregnancy with breast milk hormone concentrations. Obesity.

[62] Nilsson A., Ohlson C., Isaksson O.G., Lindahl A., Isgaard J. (1994). Hormonal regulation of longitudinal bone growth. Eur. J. Clin. Nutr..

[63] Hochberg Z., Phillip M., Youdim M.B., Amit T. (1993). Regulation of the growth hormone (GH) receptor and GH binding protein by GH pulsatility. Metabolism.

[64] Ozgurtas T., Aydin I., Turan O., Koc E., Hirfanoglu I.M., Acikel C.H., Akyol M., Erbit M.K. (2010). Vascular endothelial growth factor, basic fibroblast growth factor, insulin-like growth factor-1 and platelet-derived growth factor levels in human milk in mothers with term and preterm neonates. Cytokine.

[65] Milson S.R., Blum W.F., Gunn A.J. (2008). Temporal changes in insulin-like growth factors I and II and in insulin-like growth factors 1,2, and 3 in human milk. Horm. Res..

[66] Holton T.A., Vijayakumar V., Dallas D.C., Guerrero A., Borghese R.A., Lebrilla C.B., German J.B., Barile D., Underwood M.A., Shields D.C. (2014). Following the digestion of milk proteins from mother to baby. J. Proteome Res..

[67] Nielsen S.D., Beverly R.L., Dallas D.C. (2017). Milk proteins are predigested within the human mammary gland. J. Mammary Gland. Biol. Neoplasia.

[68] Dallas D.C., Murray N.M., Gan J. (2015). Proteolytic Systems in Milk: Perspectives on the Evolutionary Function within the Mammary Gland and the Infant. J. Mammary Gland. Biol. Neoplasia.

[69] Hamosh M. (1996). Digestion in the newborn. Clin. Perinatol..

[70] He X., McClorry S.M., Hernell O., Lőnnerdal B., Slupsky C.M. (2020). Digestion of human milk fat in healthy infants. Nutr. Res..

[71] Khaldi N., Vijayakumar V., Dallas D.C., Guerrero A., Wickramasinghe S., Smilowitz J.T., Medrano J.F., Lebrilla C.B., Shields D.C., German J.B. (2014). Predicting the important enzymes in human breast milk digestion. J. Agric. Food Chem..

[72] Dallas D.C., German J.B. (2017). Enzymes in human milk. Nestle Nutr. Inst. Workshop Ser..

[73] Enjapoori A.K., Kukuljan S., Dwyer K.M., Sharp J.A. (2019). In vivo endogenous proteolysis yielding beta-casein derived bioactive beta-casomorphin peptides in human breast milk for infant nutrition. Nutrition.

[74] Forsgård R.A. (2019). Lactose digestion in humans: Intestinal lactase appears to be constitutive whereas the colonic microbiome is adaptable. Am. J. Clin. Nutr..

[75] Lyons K.E., Ryan C.A., Dempsey E.M., Ross R.P., Stanton C. (2020). Breast Milk, a Source of Beneficial Microbes and Associated Benefits for Infant Health. Nutrients.

[76] Stinson L.F., Payne M.S., Keelan J.A. (2017). Planting the seed: Origins, composition, and postnatal health significance of the fetal gastrointestinal microbiota. Crit. Rev. Microbiol..

[77] Tanaka M., Nakayama J. (2017). Development of the gut microbiota in infancy and its impact on health in later life. Allergol. Int..

[78] Almeida C.C., Mendonça Pereira B.F., Leandro K.C., Costa M.P., Spisso B.F., Conte-Junior C.A. (2021). Bioactive compounds in infant formula and their effects on infant nutrition and health: A systematic literature review. Int. J. Food Sci..

[79] Fehr K., Moossavi S., Sbihi S., Boutin R.C.T., Bode L., Robertson B., Yonemitsu C., Field C.J., Becker A.B., Mandhane P.J. (2020). Breastmilk feeding practices are associated with the co-occurrence of bacteria in mother’s milk and the infant gut: The CHILD Cohort Study. Cell Host Microbe.

[80] Bakshi S., Paswan V.K., Yadav S.P., Bhinchhar B.K., Kharkwal S., Rose H., Kanetkar P., Kumar V., Al-Zamani Z.A.S., Bunkar D.S. (2023). A comprehensive review on infant formula: Nutritional and functional constituents, recent trends in processing and its impact on infants’ gut microbiota. Front. Nutr..

[81] CFR—Code of Federal Regulations. Title 21. 21CFR107.100. eCFR. https://www.ecfr.gov/current/title-21/chapter-I/subchapter-B/part-107/subpart-D/section-107.100.

[82] Widdowson E.M., McCance R.A. (1960). Some effects of accelerating growth. I. General somatic development. Proc. R. Soc. Lond. Ser. B Biol. Sci..

[83] Kennedy G.C. (1957). The development with age of hypothalamic restraint upon the appetite of the rat. J. Endocrinol..

[84] Kennedy G.C. (1957). The effect of age on the somatic and visceral response to overnutrition in the rat. J. Endocrinol..

[85] Widdowson E.M. (1965). Factors affecting the growth rate of laboratory animals. Food Cosmet. Toxicol..

[86] Vickers M.H., Breier B.H., Cutfield W.S., Hofman P.L., Gluckman P.D. (2000). Fetal origins of hyperphagia, obesity, and hypertension and postnatal amplification by hypercaloric nutrition. Am. J. Physiol. Endocrinol. Metab..

[87] Martorell R., Stein A.D., Schroeder D.G. (2001). Early nutrition and later adiposity. J. Nutr..

[88] Bleker L.S., de Rooij S.R., Painter R.C., Ravelli A.C., Roseboom T.J. (2021). Cohort profile: The Dutch famine birth cohort (DFBC)—A prospective birth cohort study in the Netherlands. BMJ Open.

[89] Painter R.C., Roseboom T.J., Bleker O.P. (2005). Prenatal exposure to the Dutch famine and disease in later life. Reprod. Toxicol..

[90] Heijmans B.T., Tobi E.W., Stein A.D., Putter H., Blauw G.J., Susser E.S., Slagboom P.E., Lumey L.H. (2008). Persistent epigenetic differences associated with prenatal exposure to famine in humans. Proc. Natl. Acad. Sci. USA.

[91] Taeubert M.J., Kuipers T.B., Zhou J., Li C., Wang S., Wang T., Tobi E.W., Belsky D.W., Lumey L.H., BBMRI-NL Metabolomics Consortium (2024). Adults prenatally exposed to the Dutch Famine exhibit a metabolic signature associated with a broad spectrum of common diseases. BMC Med..

[92] Fernandez-Twinn D.S., Hjort L., Novakovic B., Ozanne S.E., Saffery R. (2022). Windows of vulnerability: Consequences of exposure timing during the Dutch Hunger winter. Popul. Dev. Rev..

[93] Barker D.J.P., Eriksson J.G., Forsén T., Osmond C. (2002). Fetal origins of adult disease: Strength of effects and biological basis. Int. J. Epidemiol..

[94] Stewart C.P., Iannotti L., Dewey K.G., Michaelsen K.F., Onyango A.W. (2013). Contextualizing complementary feeding in a broader framework for stunting prevention. Matern. Child Nutr..

[95] Victora C.G., de Onis M., Hallal P.C., Blossner M., Shrimpton R. (2010). Worldwide timing of growth faltering: Revisiting implications for interventions. Pediatrics.

[96] Benyi E., Sävendahl L. (2017). The physiology of childhood growth: Hormonal regulation. Horm. Res. Paediatr..

[97] Liimatta J., Jääskeläinen J., Mäntyselkä A., Häkkinen M.R., Auriola S., Voutilainen S., Flück C.E., Lakka T.A. (2024). Accelerated early childhood growth is associated with the development of earlier adrenarche and puberty. J. Endocr. Soc..

[98] Song Y., Kong Y., Xie X., Wang Y., Wang N. (2023). Association between precocious puberty and obesity risk in children: A systematic review and meta-analysis. Front. Pediatr..

[99] Jebeile H., Kelly A.S., O’Malley G., Baur L.A. (2022). Obesity in children and adolescents: Epidemiology, causes, assessment, and management. Lancet Diabetes Endocrinol..

[100] Wing R.R., Espeland M.A., Clark J.M., Hazuda H.P., Knowler W.C., Pownall H., Unick J., Wadden T., Wagenknecht L., for the Action for Health in Diabetes (Look AHEAD) Study Group (2016). Association of Weight Loss Maintenance and Weight Regain on 4-Year Changes in CVD Risk Factors: The Action for Health in Diabetes (Look AHEAD) Clinical Trial. Diabetes Care.

[101] Wilding J.P.H., Batterham R.L., Davies M., Van Gaal L.F., Kandler K., Konakli K., Lingvay I., McGowan B.M., Oral T.K., Rosenstock J. (2022). Weight regain and cardiometabolic effects after withdrawal of semaglutide: The STEP 1 trial extension. Diabetes Obes. Metab..

[102] Aronne L.J., Sattar N., Horn D.B., Bays H.E., Wharton S., Lin W.Y., Ahmad N.N., Zhang S., Liao R., Bunck M.C. (2024). Continued Treatment with Tirzepatide for Maintenance of Weight Reduction in Adults with Obesity: The SURMOUNT-4 Randomized Clinical Trial. JAMA.

[103] Borer K.T. (2021). Why we eat too much, have an easier time gaining than losing weight, and expend too little energy: Suggestions for counteracting or mitigating these problems. Nutrients.

[104] Hinte L., Castellano-Castillo D., Ghosh A., Melrose K., Gasser E., Noé F., Masier L., Dong H., Sun W., Hoffmann A. (2024). Adipose tissue retains an epigenetic memory of obesity after weight loss. Nature.

[105] Heinig M.J., Nommsen L.A., Peerson J.M., Lonnerdal B., Dewey K.G. (1993). Energy and protein intakes of breast-fed and formula-fed infants during the first year of life and their association with growth velocity: The DARLING Study. Am. J. Clin. Nutr..

[106] Ziegler E.E. (2006). Growth of breast-fed and formula-fed infants. Nestle Nutr. Workshop Ser. Pediatr. Program.

[107] Cummings D.E., Frayo R.S., Marmonier C., Aubert R., Chapelot D. (2004). Plasma ghrelin levels and hunger scores in humans initiating meals voluntarily without time- and food-related cues. Am. J. Physiol. Endocrinol. Metab..

[108] Borer K.T., Lin P.-J., Wuorinen E. (2021). Timing of Meals and Exercise Affects Hormonal Control of Glucoregulation, Insulin Resistance, Substrate Metabolism, and Gastrointestinal Hormones, but Has Little Effect on Appetite in Postmenopausal Women. Nutrients.

[109] Borer K.T., Wuorinen E., Ku K., Burant C. (2009). Appetite responds to changes in meal content, whereas ghrelin, leptin, and insulin track changes in energy availability. J. Clin. Endocrinol. Metab..

[110] Borer K.T. (2023). Are gastrointestinal signals the principal guides to human appetite and energy balance?. Med. Res. Arch..

[111] Blundell J., Finlayson G., Axelsen M., Flint A., Gibbons C., Kvist T., Hjerpsted J.B. (2017). Effects of once-weekly semaglutide on appetite, energy intake, control of eating, food preference and body weight in subjects with obesity. Diabetes Obes. Metab..

[112] Jarvie B.C., Knight Z.A. (2022). Breaking down a gut-to-brain circuit that prevents malabsorption. Cell.

[113] Spiller R.C., Trotman I.F., Adrian T.E., Bloom S.R., Misiewicz J.J., Silk D.B. (1988). Further characterisation of the ‘ileal brake’ reflex in man—Effect of ileal infusion of partial digests of fat, protein, and starch on jejunal motility and release of neurotensin, enteroglucagon, and peptide yy. Gut.

[114] Tucker A.R., Brown H.L., Dotters-Katz S.K. (2021). Maternal weight gain and infant birth weight in women in class III obesity. Am. J. Perinatol..

[115] Oken E., Gilman M.W. (2003). Fetal origins of obesity. Obes. Res..

[116] Kalra S., Kumar A., Gupta Y. (2016). Prevention of lipohypertrophy. J. Pak. Med. Assoc..

[117] Jaquet D., Czernichow P. (2003). Born small for gestational age: Increased risk of type 2 diabetes, hypertension and hyperlipidemia in adulthood. Horm. Res..

[118] Liew G., Wang J.J., Duncan B.B., Klein R., Sharrett A.R., Brancati F., Yeh H.C., Mitchell P., Wong T.Y. (2008). Atherosclerosis risk in communities study: Low birthweight is associated with narrower arterioles in adults. Hypertension.

[119] Peng Y., Huang B., Biro F., Feng L., Guo Z., Slap G. (2005). Outcome of low birthweight in China: A 16-year longitudinal study. Acta Paediatr..

[120] Robertson R.C., Manges A.R., Finlay B.B., Prendergast A.J. (2019). The Human Microbiome and Child Growth—First 1000 Days and Beyond. Trends Microbiol..

[121] Zhou Y., Ma W., Zeng Y., Yan C., Zhao Y., Wang P., Shi H., Lu W., Zhang Y. (2021). Intrauterine antibiotic exposure affected neonatal gut bacteria and infant growth speed. Environ. Pollut..

[122] Robertson R.C., Edens T.J., Carr L., Mutasa K., Gough E.K., Evans C., Geum H.M., Baharmand I., Gill S.K., Ntozini R. (2023). The gut microbiome and early-life growth in a population with high prevalence of stunting. Nat. Commun..

[123] Johnson K.E., Hernandez-Alvarado N., Blackstad M., Heisel T., Allert M., Fields D.A., Isganaitis E., Jacobs K.M., Knights D., Lock E.F. (2024). Human cytomegalovirus in breast milk is associated with milk composition and the infant gut microbiome and growth. Nat. Commun..

